# Efficient gene editing of a model fern species through gametophyte-based transformation

**DOI:** 10.1093/plphys/kiae473

**Published:** 2024-09-12

**Authors:** Wei Jiang, Fenglin Deng, Mohammad Babla, Chen Chen, Dongmei Yang, Tao Tong, Yuan Qin, Guang Chen, Blaine Marchant, Pamela Soltis, Douglas Edward Soltis, Fanrong Zeng, Zhong-Hua Chen

**Affiliations:** MARA Key Laboratory of Sustainable Crop Production in the Middle Reaches of the Yangtze River, College of Agriculture, Yangtze University, Jingzhou 434025, China; School of Science, Western Sydney University, Penrith, NSW 2751, Australia; Xianghu Laboratory, Hangzhou 311231, China; MARA Key Laboratory of Sustainable Crop Production in the Middle Reaches of the Yangtze River, College of Agriculture, Yangtze University, Jingzhou 434025, China; Hubei Hongshan Laboratory, Wuhan 430070, China; School of Science, Western Sydney University, Penrith, NSW 2751, Australia; School of Science, Western Sydney University, Penrith, NSW 2751, Australia; School of Science, Western Sydney University, Penrith, NSW 2751, Australia; School of Tropical Agriculture and Forestry, Hainan University, Danzhou, 571737, China; MARA Key Laboratory of Sustainable Crop Production in the Middle Reaches of the Yangtze River, College of Agriculture, Yangtze University, Jingzhou 434025, China; MARA Key Laboratory of Sustainable Crop Production in the Middle Reaches of the Yangtze River, College of Agriculture, Yangtze University, Jingzhou 434025, China; Institute of Digital Agriculture, Zhejiang Academy of Agricultural Science, Hangzhou 310021, China; Department of Biology, University of Missouri—St. Louis, St. Louis, MO 63121, USA; Department of Biology, University of Florida, Gainesville, FL 32611, USA; Department of Biology, University of Florida, Gainesville, FL 32611, USA; MARA Key Laboratory of Sustainable Crop Production in the Middle Reaches of the Yangtze River, College of Agriculture, Yangtze University, Jingzhou 434025, China; School of Science, Western Sydney University, Penrith, NSW 2751, Australia; Hawkesbury Institute for the Environment, Western Sydney University, Penrith, NSW 2751, Australia

## Abstract

The clustered regularly interspaced short palindromic repeats (CRISPR)/CRISPR-associated nuclease (Cas) system allows precise and easy editing of genes in many plant species. However, this system has not yet been applied to any fern species through gametophytes due to the complex characteristics of fern genomes, genetics, and physiology. Here, we established a protocol for gametophyte-based screening of single-guide RNAs (sgRNAs) with high efficiency for CRISPR/Cas9-mediated gene knockout in a model fern species, *Ceratopteris richardii*. We utilized the *C. richardii ACTIN* promoter to drive sgRNA expression and the enhanced CaMV 35S promoter to drive the expression of *Streptococcus pyogenes* Cas9 in this CRISPR-mediated editing system, which was employed to successfully edit a few genes, such as *Nucleotidase/phosphatase 1* (*CrSAL1*) and *Phytoene Desaturase* (*CrPDS*), which resulted in an albino phenotype in *C. richardii*. Knockout of *CrSAL1* resulted in significantly (*P* < 0.05) reduced stomatal conductance (*g_s_*), leaf transpiration rate (*E*), guard cell length, and abscisic acid (ABA)-induced reactive oxygen species (ROS) accumulation in guard cells. Moreover, *CrSAL1* overexpressing plants showed significantly increased net photosynthetic rate (*A*), *g_s_*, and *E* as well as most of the stomatal traits and ABA-induced ROS production in guard cells compared to the wild-type (WT) plants. Taken together, our optimized CRISPR/Cas9 system provides a useful tool for functional genomics in a model fern species, allowing the exploration of fern gene functions for evolutionary biology, herbal medicine discovery, and agricultural applications.

## Teaser

Gametophyte-based screening of single-guide RNAs with high efficiency for gene editing in *Ceratopteris richardii* provides a useful tool in functional genomics with widespread applications in ferns.

## Introduction

First appearing in the fossil record around 360 million years ago (MYA), true ferns form the second largest vascular plant lineage after angiosperms with more than 10,500 species (https://www.worldfloraonline.org/). These numerous species have been instrumental in shaping plant biodiversity and numerous ecosystems on Earth, resulting in a breadth of adaptations and innovations that are fascinating for research in genomics, evolution, ecology, molecular biology, and physiology (Cai et al. 2021; Marchant et al. 2022). Compared to other vascular plants, distinct genes (e.g. *Phenolic Acid Decarboxylases*, *Aerolysin-like*, and *12-oxo-Phytodienoic Acid*) might protect ferns from biotic (Pennisi 2023; Wei et al. 2023) and abiotic stresses (Yan et al. 2019). Many fern species are used in traditional medicine for treating fevers, relaxing muscles, and relieving pain due to the active chemical compounds they produce (Cao et al. 2017; Kumar et al. 2023; Pohthmi and Sharma 2023).

The CRISPR/Cas9 and CRISPR/Cas12 have been widely used in plant molecular research due to its simplicity, versatility, and efficiency for gene editing (Xie et al. 2015; Endo et al. 2019; Wang et al. 2020; Cardi et al. 2023; Fan et al. 2023; Bai et al. 2024; Pacesa et al. 2024). The cellular repair of CRISPR/Cas9-mediated double-strand breaks by non-homologous end joining using sgRNA and Cas9 nuclease can lead to the modification of genes (Wang et al. 2018; Wang et al. 2020; Ahmar et al. 2024). The ability to reprogram CRISPR/Cas9 with engineered sgRNA to target any gene of interest allows plant scientists to develop new plant varieties with desired traits without the regulatory complication of genetically modified organism (GMO) (He et al. 2022; Cardi et al. 2023; Pacesa et al. 2024). For instance, CRISPR/Cas9-mediated inactivation significantly enhanced grain weight in rice (*Oryza sativa*) by targeting *Grain Width/Weight* (*OsGW5*) (Liu et al. 2017a, 2017b, 2017c) and *MADS-BOX TRANSCRIPTION FACTOR 1* (*OsMADS1*) (Wang et al. 2024), production of low-gluten wheat (*Triticum aestivum*) through editing the *α-gliadin* gene array (Sánchez-León et al. 2018), and powdery mildew resistance of tomato (*Solanum lycopersicum*) (Nekrasov et al. 2017). In the past decade, CRISPR/Cas technology has been successfully utilized to modify more than 130 green plant species based on a recent review (Cardi et al. 2023), including 110 angiosperms (mostly agricultural and horticultural crops with significant economic values) (Kis et al. 2019; Wang et al. 2023a, 2023b), and 7 gymnosperms (Ren et al. 2021; Ye et al. 2023), 1 fern (Xiang and Li 2024), 3 mosses (Tansley et al. 2023; Yuan et al. 2023; Tavernier et al. 2024), and 12 algae (Belshaw et al. 2023; Patel et al. 2023; Zhang et al. 2023), without any species of lycophytes.

*Ceratopteris richardii* is a fast-growing, small, tropical homosporous fern that has been used for decades as the model fern species (Marchant et al. 2019). Genetic transformation has been performed in *C. richardii* for functional genomics (Plackett et al. 2014, 2015) such as discovering the roles of genes in sex determination (Youngstrom et al. 2019), genome structure, developmental biology (Plackett et al. 2018; Geng et al. 2022), hybridization and reproductive barriers (Youngstrom et al. 2022; Withers et al. 2023), and apogamy (Bui et al. 2017). In addition, the molecular function of some *C. richardii* genes has been studied through RNA interference (RNAi) (Plackett et al. 2018; Withers et al. 2023), gene editing (Xiang and Li 2024), and overexpression methods (Youngstrom et al. 2022). While the genetic transformation of fern gametophytes as the explant usually has a low success rate, it should be noted that the majority of these methods were developed and optimized according to the well-established protocols targeting to angiosperm flowers, immature embryos, and calli (Bui et al. 2015; Bui et al. 2017). An efficient gene editing protocol for fern species has not been developed through gametophytes, but an efficient and fast verification system in *C. richardii* will facilitate the analysis of gene function in ferns (Frangedakis et al. 2023).

Nucleotidase/phosphatase SAL1, also known as FIERY1 (FRY1) (Ishiga et al. 2017), has a dual enzymatic activity of nucleotidase and inositol phosphatase, which functions largely in responses to abiotic stresses through inositol signaling and nucleotide metabolism (Jia et al. 2019). Transient silencing of *SAL1* and loss-of-function mutants led to enhanced drought tolerance in *T. aestivum* (Manmathan et al. 2013; Abdallah et al. 2022) and Arabidopsis (*Arabidopsis thaliana*) (Wilson et al. 2009; Estavillo et al. 2011), while *OsSAL1* overexpression plants were severely impaired in drought tolerance of rice (Liu et al. 2023). Additionally, *GhSAL1* negatively modulated cold tolerance via inositol 1,4,5-triphosphate-Ca^2+^ signaling pathway in cotton (*Gossypium hirsutum*) (Shen et al. 2023). Our previous study showed that *C. richardii* SAL1 (CrSAL1) and its byproduct 3′-phosphoadenosine-5′-phosphate (PAP) function as chloroplast stress signals and participated in the abscisic acid (ABA) signaling pathway for drought response and stomatal regulation (Zhao et al. 2019), but *CrSAL1* was not functionally verified through genetic engineering in *C. richardii*.

Here, we established an efficient gene editing platform for *C. richardii* transformation using gametophytes. We improved the targeting and editing efficiency of sgRNAs for an optimized *Agrobacterium* (*Agrobacterium tumefacien*s)-mediated CRISPR/Cas9 system via the successful editing of *CrSAL1* (*Ceric.25G052000.1*) and *CrPDS* (*Ceric.08G066500.1*) in *C. richardii*. Knockout and overexpression of *CrSAL1* resulted in distinctive phenotypes in gas exchange parameters and stomatal traits in the transgenic plants compared to those in the WT. Our study suggests that the CRISPR/Cas system and the potentially expanded toolkit for gene editing in ferns will facilitate more rapid gene discovery and functional validation for herbal medicine, evolutionary biology, and agricultural applications.

## Results

### Selection of fern species and developmental stages for transformation

Several reference genome of ferns have been assembled in recent years, including *Azolla filiculoides* (0.75 Gb, *n* = 22), *Salvinia cucullata* (0.26 Gb, *n* = 9) (Li et al. 2018), *Alsophila spinulosa* (6.27 Gb, *n* = 69) (Huang et al. 2022), *Adiantum capillus*-*veneris* (4.83 Gb, *n* = 30) (Fang et al. 2022), *C. richardii* (7.46 Gb, *n* = 39) (Marchant et al. 2022), and *Marsilea vestita* (1.0 Gb, *n* = 20) (Rahmatpour et al. 2023) (Table 1). These high-quality genome sequences enable future research into the functional genomics and applications of ferns (Chen 2022; Kinosian and Wolf 2022; Frangedakis et al. 2023). In the available transformation methods, particle bombardment and *Agrobacterium*-mediated stable transformation have been successfully applied to *C. richardii* (Plackett et al. 2014; Bui et al. 2015; Xiang and Li 2024) and *Pteris vittata* (Indriolo et al. 2010; Muthukumar et al. 2013). These robust transformation methods have paved the way for the development of gene editing in ferns. While *P. vittata* lacks the necessary genomic information for extensive genetic manipulation (Petlewski and Li 2019), the recent publication of the *C. richardii* genome led us to select *C*. *richardii* as the most suitable fern species for establishing a gene editing protocol.

**Table 1.** Overview of overexpression, RNAi, CRISPR/Cas9 in fern species

			Transformation methods				
Species	Genome size	Chromosome number	RNAi	DNAi	*Agrobacterium*-mediated	Particle bombardment	CRISPR/Cas9
*Ceratopteris richardii*	7.46 Gb, Marchant et al. 2022	*n* = 39	Stout et al. 2003	Rutherford et al. 2004	Muthukumar et al. 2013; Bui et al. 2015	Rutherford et al. 2004; Plackett et al. 2014, 2015	Xiang and Li 2024, This study
*Azolla filiculoides*	0.75 Gb, Li et al. 2018	*n* = 22	–	–	–	–	–
*Salvinia cucullata*	0.26 Gb, Li et al. 2018	*n* = 9	–	–	–	–	–
*Marsilea vestita*	1.0 Gb, Rahmatpour et al. 2023	*n* = 20	Klink and Wolniak 2001	–	–	–	–
*Adiantum capillus-veneris*	4.83 Gb, Fang et al. 2022	*n* = 30	–	Kawai-Tooyoka et al. 2004	–	Kawai et al. 2003	–
*Alsophila spinulosa*	6.27 Gb, Huang et al. 2022	*n* = 69	–	–	–	–	–
*Pteris vittata*	–	*n* = 29	Indriolo et al. 2010	–	Muthukumar et al. 2013	Indriolo et al. 2010; Muthukumar et al. 2013	–

**Figure 1.**
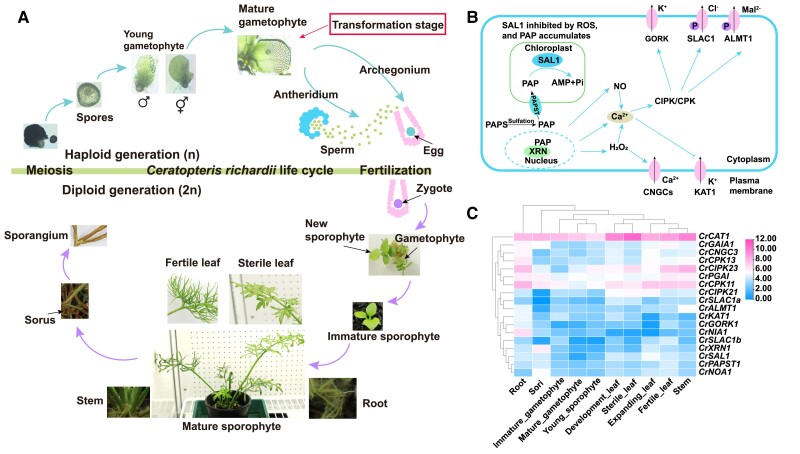
The lifecycle, proposed model and gene expression of SAL1-PAP retrograde signaling in a model fern species *Ceratopteris richardii*. **A)** The lifecycle of *C. richardii*. Images are not to scale. After meiosis produces haploid spores, the haploid gametophyte (n) starts generation. Spores germinate into either male gametophytes or hermaphrodite that produce gametes (sperm and egg) through mitosis. After fertilization, the diploid sporophyte (2n) generation begins as a zygote that generates into an embryo with its first root and leaf, initially dependent on the gametophyte. In the vegetative stage, the independent sporophyte produces sterile leaves (trophophyll), followed by increasingly dissected and lobed fronds. In the reproductive stage, the fertile leaves (sporophyll) of the sporophyte develop sporangia through meiosis on their undersides, closing the cycle. Arrows: order, blue indicating haploid gametophyte and red indicating diploid sporophyte stages. **B)** Model of SAL1-PAP retrograde signaling in plant. SAL1 is a key stress sensor through regulating retrograde signaling. Stress-induced ROS accumulation is sensed by SAL1 via redox-mediated inhibition of SAL1 activity, allowing PAP accumulation. PAPST imports cytosolic PAP in and out of the chloroplast to inhibit XRNs in the cytosol and nucleus. Many Ca^2+^ signaling proteins such as CPKs and CIPKs could be mediated through PAP-XRN-modulated retrograde signaling for the activation of ion channels through phosphorylation. Arrows: activation, lines with bars: inhibition. **C)** Expression of key genes associated with SAL1 pathway in diverse tissues such as immature gametophyte, mature gametophyte, young sporophyte, expanding leaf, development leaf, fertile leaf, sterile leaf, stem, root, sori. It was shown using the average TPM (transcripts per million) value with three biological replicates. The color scale for log_2_ TPM values is showed in the right, red indicating high expression and blue indicating low expression. SAL1, 3′(2′),5′-bisphosphate nucleotidase 1; PAP, 3′-phosphoadenosine 5′-phosphate; XRN, exoribonuclease; GORK, guard cell outward rectifying K^+^ channel; SLAC1, S-type anion channel 1; ALMT1, aluminum-activated malate transporter 1; CIPK, CBL-interacting serine/threonine-protein kinase; CPK, calcium-dependent protein kinase; CNGC, cyclic nucleotide-gated ion channel; GAIA, GAMETOPHYTES ABA INSENSITIVE ON A_CE_1; CAT, catalase peroxidase; PAPST, sulfate donor 3′-phosphoadenosine 5′-phosphosulfate transporter; NOA, oxide-associated1; NIA, nitrate reductase; XRN, exoribonuclease.

Unlike seed plants, homosporous ferns, including *C. richardii,* possess morphologically and developmentally distinct free-living haploid gametophytes and diploid sporophytes (Fig. 1A). The germination of a haploid spore to produce a photosynthetic thallus initiates the gametophytic generation. Hormonal sex determination of C. *richardii* differentiates individual gametophytes into distinct male or hermaphrodite sexes (Conway and Di Stilio 2020). Archegonia (female gametangia) and antheridia (male gametangia) develop to produce eggs and motile sperm, respectively (Fig. 1A). Only one archegonium is fertilized, resulting in a single diploid zygote per gametophyte. This first step in the diploid sporophyte generation is crucial for genetic transformation (Muthukumar et al. 2013; Bui et al. 2015; Bui et al. 2017). Extrapolating from the successful transformation of the liverwort *Marchantia polymorpha* (Ishizaki et al. 2008) and *C. richardii* (Bui et al. 2015) gametophytes via *Agrobacteria*, we developed an *Agrobacterium*-mediated mature gametophyte system for gene editing (knockout) in *C. richardii*. The gametophyte dies after the first sporophyte leaves are produced. The sporophyte produces a vegetative shoot from which fronds and roots emerge. The first frond produced is a sterile leaf, but later fronds become fertile leaves. The life cycle of *C. richardii* is completed with the production of haploid spores (Fig. 1).

### Identification and cloning of U6 promoter and *Actin* promoter from *C. richardii*

Guide RNAs for genome editing have been produced using a range of U6 promoters (Xie et al. 2015; Kor et al. 2022) in monocots (Kis et al. 2019; Wang et al. 2023a, 2023b), eudicots (Kis et al. 2019; Wang et al. 2023a, 2023b), gymnosperms (Ren et al. 2021; Ye et al. 2023), and mosses (Tansley et al. 2023; Yuan et al. 2023; Tavernier et al. 2024). We identified seven *U6 small nuclear ribonucleoprotein* genes (*Ceric.17G074700*, *Ceric.33G040100*, *Ceric.09G088700*, *Ceric.02G026900*, *Ceric.1Z290000*, *Ceric.03G070800*, and *Ceric.03G071600*) in the *C. richardii* genome (https://phytozome-next.jgi.doe.gov/info/Crichardii_v2_1), which showed high expression in gametophyte, leaf, stem, and root (Supplementary Fig. S1A). However, the promoters of these *C. richardii* genes do not contain the upstream sequence element (USE) and TATA elements, which are the typical structural properties of the Pol III promoters (Kor et al. 2022). Furthermore, we identified three putative orthologs of *AtU6* in *C. richardii,* including *CrU6-1* (*Ceric.13G012200*), *CrU6-2* (*Ceric.13G012300*), and *CrU6-3* (*Ceric.1Z176900*). Their promoters possess the USE and TATA elements (Supplementary Fig. S1, B and S2), which could be used to drive sgRNA in the CRISPR/Cas9 system of ferns.

Previous studies showed that a single Pol II promoter (either constitutive or inducible) can also achieve effective gene editing (Hassan et al. 2021; Cardi et al. 2023) by driving gRNA/crRNA in *O. sativa* (Tang et al. 2016; Ren et al. 2019; Fan et al. 2024), *T. aestivum* (Luo et al. 2021), *G. hirsutum* (Hui et al. 2024), *Hordeum vulgare*, *S. lycopersicum*, *Medicago truncatula* (Čermák et al. 2017), and the diatom *Phaeodactylum tricornutum* (Taparia et al. 2022). The *Actin* promoter isolated from *P. vittata* was able to function efficiently in both *P. vittate* and *C. thalictroides* (Muthukumar et al. 2013). Therefore the upstream of the *CrActin* was isolated and considered as the putative promoter (Supplementary Fig. S2A), which was instead of the OsU3 promoter in pRGEB32 (Xie et al. 2015) to drive the expression cassettes of sgRNA (Luo et al. 2021). The Cas9 protein was also reported to be driven by the enhanced cauliflower mosaic virus (CaMV) 35S promoter (Li et al. 2013; Sugano et al. 2014; Awasthi et al. 2021; Cui et al. 2021). Therefore, the native maize (*Zea mays*) ubiquitin promoter (ZmUbi) promoter in the original construct pRGEB32 was replaced by the enhanced 35S promoter (Supplementary Fig. S2B), which was designated as pRGEB32-CrActin. Moreover, the high activity of CrActin and low activity of CrU6-2 promoters were identified by luciferase (LUC) and β-glucuronidase (GUS) assays (Supplementary Fig. S2, E and F). Thus, the CrActin promoter was used in further experiments.

### An efficient Agrobacterium-mediated transformation of *C. richardii* using hygromycin selection

To get positive transformants with gene editing or overexpression, the transformation protocol of *C. richardii* was optimized through adjusting the time for enzyme treatment, co-incubation, and the concentrations with hygromycin for positive selection (Table 2). Subsequently, *CrSAL1* was selected to establish the *Agrobacterium*-mediated transformation of *C. richardii*. SAL1-PAP retrograde signaling involves stomatal opening and closure through ROS, Ca^2+^, and nitric oxide (NO) pathways and ion channels (Pornsiriwong et al. 2017; Zhao et al. 2019) (Fig. 1B). Here, we found that key components of the SAL1-PAP retrograde signaling pathway such as *CrSAL1*, *CrCAT1* (*Catalase Peroxidase* 1), ion channels [*CrALMT1* (*Aluminum-activated Malate Transporter 1*), *CrCNGC3* (*Cyclic Nucleotide-gated Ion Channel 1*)] and protein kinases [*CrCIPK11* (*CBL-interacting Serine/threonine-Protein Kinase 11*), *CrCIPK23*] displayed high levels of expression in most of the tissues, particularly in leaves (Fig. 1C).

**Table 2.** Factors affecting the efficiency of genetic transformation of the fern species *C. richardii*

Construct	Total number of gametophytes	OD value	Enzyme treatment time	Co-incubated *Agrobacterium* time	Hygromycin selection	Transformants	Transformation efficiency	Tested T0 seedling	Mutated T0 seedling number	Ratio
Untransformed	215	0.4	15 min	15 min	10 mg/L	0	0%	–	–	–
*SAL1-OE*	893	0.4	15 min	15 min	10 mg/L	8	0.90%	–	–	–
*SAL1-OE*	537	1.2	15 min	15 min	10 mg/L	6	1.12%	–	–	–
*SAL1-OE*	768	0.8	2 h	1 h	5, then 20 mg/L	73	9.51%	–	–	–
*CRY4-OE*	194	0.8	2 h	1 h	5, then 20 mg/L	23	11.68%	–	–	–
*YSL-OE*	351	0.8	2 h	1 h	5, then 20 mg/L	26	7.41%	–	–	–
*GRF-OE*	159	0.8	2 h	1 h	5, then 20 mg/L	14	8.81%	–	–	–
*SAL1-KO-target1*	210	0.8	2 h	1 h	5, then 20 mg/L	8	3.33%	8	1	12.50%
*SAL1-KO-target2*	210	0.8	2 h	1 h	5, then 20 mg/L	8	3.33%	8	2	25.00%
*PDS-KO*	212	0.8	2 h	1 h	5, then 20 mg/L	10	4.72%	10	2	20.00%
*PDS-KO-target2*	212	0.8	2 h	1 h	5, then 20 mg/L	10	4.72%	0	0	0%
*CRY4-KO*	526	0.8	2 h	1 h	5, then 20 mg/L	28	5.32%	–	–	–
*YSL-KO*	468	0.8	2 h	1 h	5, then 20 mg/L	21	4.49%	–	–	–

**Figure 2.**
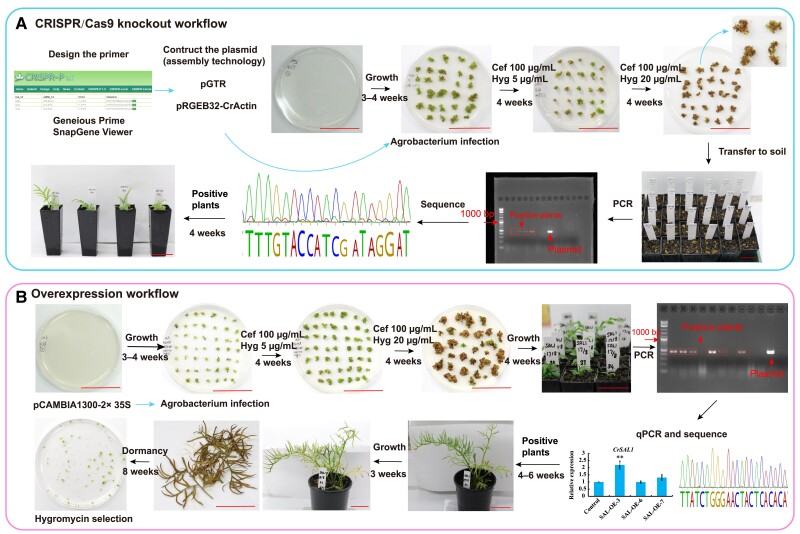
The workflow of gene editing and gene overexpression in *C. richardii*. **A)** Gene knockout; **B)** gene overexpression, bar = 5 cm. The sgRNAs were designed through CRISPR-P 2.0 (http://crispr.hzau.edu.cn/cgi-bin/CRISPR2/SCORE). Overexpression and CRISPR/cas9 constructs were generated utilizing the assembly technology. After *Agrobacterium* infection, gametophytes were grown at MS media with 5 mg/L of hygromycin and 100 mg/L of cefuroxime for 30 days. Then, the sporophytes were transferred to MS media supplemented with 100 mg/L cefotaxime and 20 mg/L hygromycin for another 30 days. The regeneration sporophytes were then transplanted to pots containing a premium potting mix for PCR and qPCR analysis. The images have been reused for the same treatment. Values are means of three biological replicates ± SE. Asterisks indicate significant differences compared with the WT plants (**P < 0.01). Arrows: order; Cef, cefotaxime; Hyg, hygromycin.

The core competence of CRISPR/Cas9 system contains the expression cassettes of gRNA and the SpCas9 nuclease (Supplementary Fig. S3A). The pRGEB32-CrActin (Supplementary Fig. S3C), and pCAMBIA1300 (Supplementary Fig. S3D) were employed for gene editing and overexpression in *C. richardii*, respectively. The transformation construct used for stable overexpression transformation was pCAMBIA1300-2× 35S, which carries the *hygromycin phosphotransferase* (*HPT*) gene for the selection of positive transgenic plants. After 72 h of co-incubation with *Agrobacteria*, transformed gametophytes were selected on Murashige and Skoog (MS) media supplemented with cefotaxime (100 mg/L) and hygromycin (5 mg/L and 20 mg/L) to kill the *Agrobacteria* and select the transformants, respectively (Fig. 2).

We found that the gametophytes are unable to reproduce and survive for long periods under the suggested MS media with 20 mg/L hygromycin. To increase the regeneration and survival rate of the transformed gametophytes, we assayed a range of hygromycin concentrations and selected 5 mg/L (Supplementary Fig. S4, A and B) as the preferred concentration to obtain more regenerated gametophytes with normal morphology and reproduction (Fig. 2). The sporophytes were then transferred to MS media supplemented with 100 mg/L cefotaxime and 20 mg/L hygromycin for another 30 days. The highest regeneration rate for stable transformation was achieved by 2 h treatment with 1.5% (w/v) cellulase before *Agrobacterium* co-incubation. We observed that the sporophyte survival rate was slightly increased by *Agrobacterium* co-incubation time with 1.5% cellulase for 1 h (Table 2). Therefore, a combination of digestion with 1.5% cellulase and selection with 100 mg/L cefotaxime and 5/20 mg/L hygromycin was employed in our experiments. Interestingly, regeneration rarely occurs in a 1:1 stoichiometry, and a cluster of diverse regenerated gametophytes were developed from a gametophyte inoculated with *Agrobacterium*. The regenerated sporophytes were then transplanted to pots containing a premium potting mix for further analysis.

### Molecular analysis of *CrSAL1* overexpression plants

Nearly 10% of treated gametophytes survived on MS media supplemented with 20 mg/L hygromycin (Fig. 2B). We obtained 87 *CrSAL1* overexpression plants that survived under hygromycin selection, but half of the plants failed to develop normally and complete the life cycle (Supplementary Fig. S5A). Positive transgenic plants were screened by PCR with a 456-bp PCR product using the DNA as template and hygromycin primers targeting the *HPT* gene (Supplementary Fig. S5B). In total, we obtained and verified 15 transgenic *C. richardii* individuals with relatively higher expression of *CrSAL1* (Supplementary Fig. S5). The transformation efficiency was calculated according to the number of successfully developed transgenic sporophytes divided by the total gametophytes used in transformation and multiplied by 100 (Bui et al. 2017), resulting in an efficiency ranging from 3.3% to 11.68% across those tested genes (Table 2).

### Screening of gene knockout lines of CrPDS and CrSAL mediated by CRISPR/Cas9

**Figure 3.**
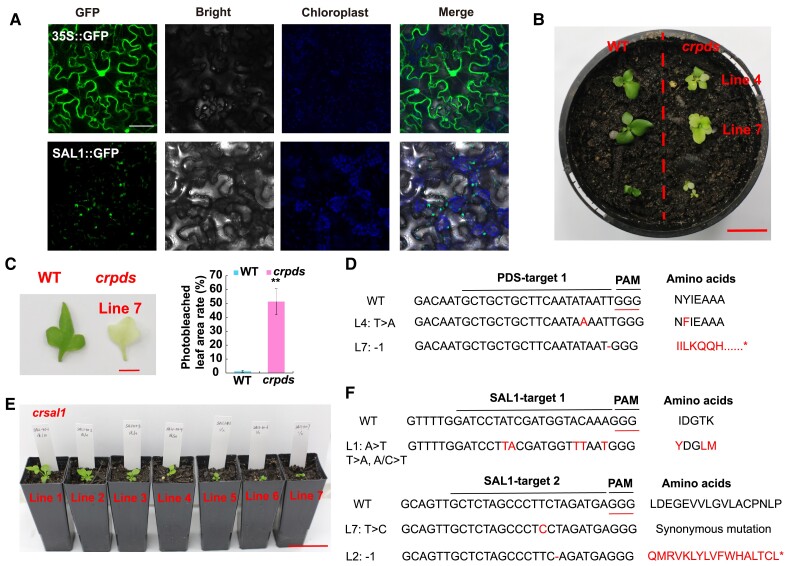
Phenotype and sequences of the editing types of *CrPDS* and *CrSAL1* transgenic plants. **A)** Subcellular localization patterns of CrSAL1 in tobacco leaves, bars = 60 *μ*m. Phenotypes of WT and transgenic plants [*CrPDS***(B)**, bars = 2 cm; **C)**, bars = 5 cm)]. **D)** Sanger sequencing of the editing types in *CrPDS* transgenic plants. **E)** Phenotypes of *crsal1* plants, bars = 5 cm. **F)** Sanger sequencing of the editing types in *CrSAL1* transgenic plants. Red symbol, mutated nucleotides or amino acids. Values are means of 3 biological replicates ± SE. Asterisks indicate significant differences compared with the WT plants (**P < 0.01).

The subcellular localization of green fluorescent protein (GFP) fusion construct in the tobacco (*Nicotiana benthamiana*) epidermis showed that GFP alone was found in the nuclei, cytoplasm, and membranes. However, we found GFP fluorescence of CrSAL1 overlaps with the chloroplast fluorescence (Fig. 3A). The results indicate that CrSAL1 may function in chloroplast retrograde signaling and stomatal regulation, which is similar to those in *A. thaliana* (Xiong et al. 2001; Estavillo et al. 2011).

After the successful establishment of the *Agrobacterium*-mediated stable transformation method for the overexpression gene of interest in *C. richardii* using gametophytes as the explant, the pipeline was employed to generate the gene editing lines with CRISPR/Cas9 system in *C. richardii*. Loss-of-function of *Phytoene desaturase* (*PDS*) leads to photobleaching phenotypes in varied plant species (Awasthi et al. 2021), which was widely employed as a visible marker in developing the protocol for knocking out genes of interest (Ma et al. 2019). To introduce mutations into the *CrPDS*, two independent 20-bp sequences with NGG trinucleotide in their 3′-regions targeting were synthesized and inserted into the gRNA expression cassette of the pRGEB32-CrActin vector. We obtained 18 *CrSAL1* and *CrPDS* CRISPR/Cas9 plants through screening with hygromycin (Supplementary Fig. S6, Fig. 3, Table 2). The positively transformed plants showed the expected photobleached leaf phenotype (Fig. 3, B and C). The fragments containing the target sites were amplified with high-fidelity polymerase and purified. Sanger sequencing (ABI 3500 Genetic Analyzer, Thermofisher, Waltham, MA, USA) was performed. Primers used for sequencing were listed in Supplementary Table S1. Sequence analysis determined that the editing efficiency of the *CrPDS* and *CrSAL1* target site in the transgenic plants ranged from 0% to 25%, although the transformation efficiency of gene knockout ranged from 3.33% to 4.72%. Both of replacement and deletion could be found in the mutant lines (Fig. 3, D and F). These results suggest that the pRGEB32-CrActin we generated in this study could be employed for editing genes of interest in *C. richardii* (Table 2).

### Physiological evaluation of SAL1 overexpression and knockout *C. richardii* plants

**Figure 4.**
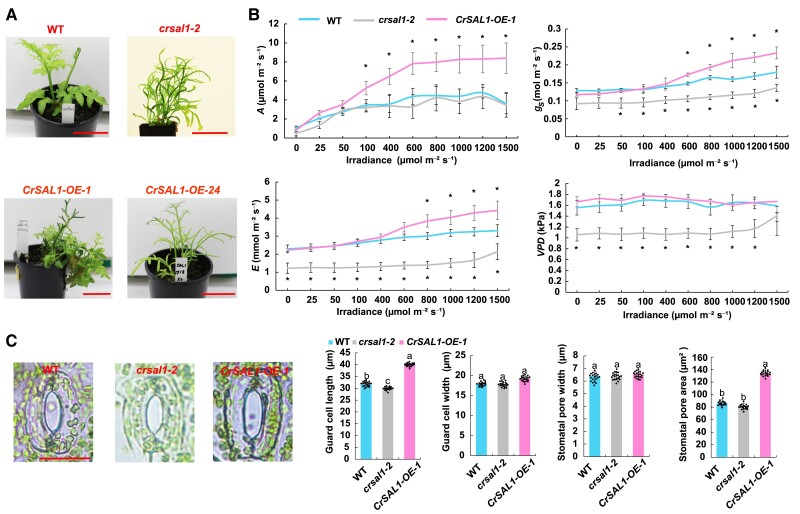
Photosynthesis and stomatal traits of *CrSAL1* gene editing and overexpression lines. **A)** Phenotype and gas exchange parameters of WT, *SAL1-OE*, and *crsal1* plants, bars = 5 cm. **B)** Net CO_2_ assimilation (*A*), leaf transpiration rate (*E*), stomatal conductance (*g_s_*), and vapor pressure deficit (*VPD*) of transgenic and WT plants (*n* = 6). **C)** Stomatal parameters of transgenic and WT plants, bar = 20 *μ*m. Values are means of three biological replicates ± SE with 20 to 30 stomata. Asterisks indicate significant differences compared with the WT plants (*P < 0.05). Different letters indicate significant differences compared with the WT plants (*P* < 0.05). The SPSS 26.0 software (IBM, USA) was employed to perform the analysis of variance (ANOVA) and means were compared by Duncan's multiple range tests.

We overexpressed *CrSAL1* in *C. richardii* and obtained 15 individuals with relatively higher expression of *CrSAL1* (Supplementary Fig. S5), but only four individuals (Line 1, 13, 21 and 24) completed the life cycle (Fig. 4A). Overexpression *CrSAL1-OE-1* (Line 1) in *C. richardii* significantly increased the net CO_2_ assimilation (*A*), leaf transpiration rate (*E*), and stomatal conductance (*g_s_*) under high light intensity compared to the WT across light intensity from 0 to 1500 *μ*mol m^−2^ s^−1^. Interestingly, the CRISPR/Cas9 knockout mutant *crsal1-2* displayed significantly lower *g_s_*, *E*, and vapor pressure deficit (*VPD*) compared to the WT (Fig. 4B).

Stomata are important for plants to respond to environmental conditions (Hetherington and Woodward 2003; Chen et al. 2017; Jiang et al. 2024). Under the control conditions, guard cell length and stomatal pore area in the *CrSAL1-OE-1* lines were significantly increased compared to the WT plants, on average, by 25.3% and 55.0%, respectively (Fig. 4C). In contrast, the *crsal1-2* knockout mutant showed a slight decrease in the guard cell length compared to the WT (Fig. 4C).

**Figure 5.**
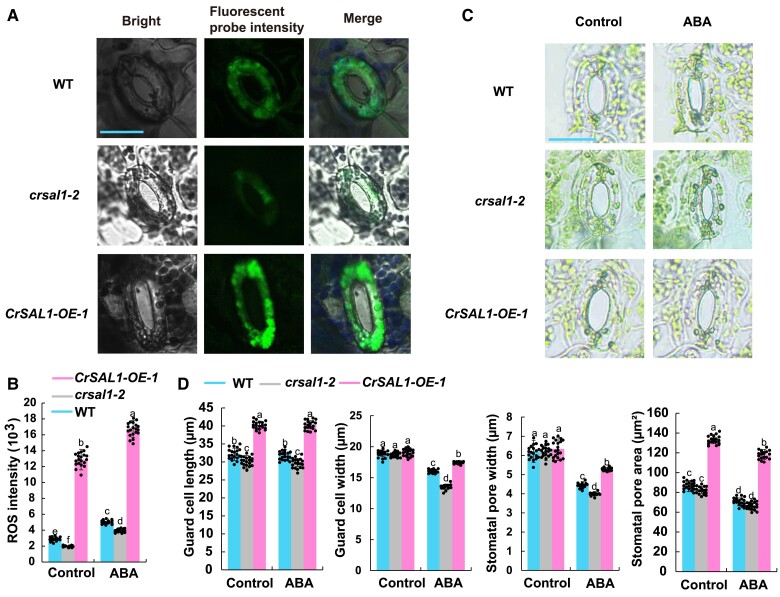
Effects of gene editing and overexpression *CrSAL1* on reactive oxygen species (ROS) and ABA response of fern plants. **A)** Confocal images of ROS fluorescence in guard cells of the *SAL1* CRISPR/Cas9 and overexpression plants, bar = 20 *μ*m. **B)** Fluorescent ROS intensity in guard cells of *C. richardii* under control condition and 50 μμ ABA treatment for 60 min. **C)** ABA-induced stomatal close in transgenic and WT plants, bar = 20 *μ*m. **D)** Stomatal traits of *C. richardii* in the control and 50 μμ ABA treatment for 60 min. The ame stomata were used before and after ABA treatment. Values are means of three biological replicates ± SE with 20 to 30 stomata. Asterisks indicate significant differences compared with the WT plants in the control or ABA treatment (*P < 0.05; **P < 0.01). The SPSS 26.0 software (IBM, USA) was employed to perform the analysis of variance (ANOVA) and means were compared by Duncan's multiple range tests.

Furthermore, *CrSAL1-OE-1* plants exhibited significantly higher ROS levels in guard cells than WT under the control conditions, whereas the production of ROS in *crsal1-2* plants was significantly decreased (Fig. 5, A and B), similar to the results of previous studies on the mutants of *SAL1* gene such as *altered ascorbate peroxidase 2* (*APX2*) *expression 8* (*alx8*) and *onset of leaf death 101* (*old101*) in *A. thaliana* (Estavillo et al. 2011; Shirzadian-Khorramabad et al. 2022). SAL1 was reported to be important for ABA signaling in response to environmental conditions (Pornsiriwong et al. 2017; Zhao et al. 2019). Thus, we also performed the stomatal assay with ABA treatment in the WT and transgenic plants. After ABA treatment, the stomatal pore area of WT, *crsal1-2*, *CrSAL1-OE-1* was significantly reduced by 18.1%, 22.3%, and 11.6%, respectively (Fig. 5D). Therefore, *crsal1-2* mutant displayed ABA-sensitive stomatal phenotype (Fig. 5, C and D), which is consistent with the previous results that *sal1*-*8* (Pornsiriwong et al. 2017) and *fry1* (Xiong et al. 2001) were more sensitive to ABA in *A. thaliana*. Moreover, *CrSAL1-OE-1* showed a less ABA-sensitive stomatal phenotype (Fig. 5, C and D) similar to ectopic expression cotton *GmSAL1* in *A. thaliana* with reduced stomatal response to ABA (Ku et al. 2013). Furthermore, ABA treatment increased the ROS level of guard cell in WT, *crsal1-2*, *CrSAL1-OE-1* plants (Fig. 5B). In summary, we demonstrated gene editing in *C. richardii* through gametophytes by editing two important genes and analyzed the function of *CrSAL1.*

## Discussion

### Gene editing for gene functional verification in a fern through gametophytes

CRISPR/Cas genome editing has been applied to a variety of plant species to enhance disease resistance and abiotic stress tolerance (Deng et al. 2022). In this study, we established an efficient gene editing (knockout) and overexpression method for the transformation of gametophytes in *C. richardii.* We successfully overexpressed *CrSAL1* and other genes in *C. richardii* gametophytes by adjusting the hygromycin concentration (Bui et al. 2015), optical density (OD) value of *Agrobacterium*, age of gametophytes, and the time of enzyme treatment on gametophytes and co-cultivation with *Agrobacterium* (Table 2, Fig. 2). This optimized protocol enabled the successful establishment of the stable *Agrobacterium*-mediated CRISPR/Cas9 transformation in *C. richardii*. This system can edit genes with high efficiency in *C. richardii*, which was verified with *CrPDS* and other genes. In this study, the albino phenotype of *PDS* mutants (Fig. 3) were not as pronounced as in tobacco and rice. It could be attributed to higher number of chloroplasts in ferns (Cai et al. 2021), the functional redundancy of four *PDS* genes in the *C. richardii* genome (Wang et al. 2018), and potential chimerism (Nishitani et al. 2016; Zhang et al. 2021; Li et al. 2023). Thus, the *PDS* mutants showed the albino phenotype (Ma et al. 2019), chimeras of variegated phenotype (Awasthi et al. 2021), and pale green chimeric phenotype (Wang et al. 2018). Genetic transformation has been conducted in *C. richardii* with *Agrobacterium* infection (Bui et al. 2015) and particle bombardment (Plackett et al. 2014, 2015). Gene editing of *C. richardii* was performed through particle bombardment using callus induction followed by sporophyte regeneration (Xiang and Li 2024). In the study, the OsU3 and ZmUbi promoters of pRGEB32 plasmid were replaced by native promoters of *CrU3* and *CrActin* (*Ceric.08G028400*), respectively. However, the main advantage of our method is applying gene editing on gametophytes, which is easier and faster with relatively high transformation efficiency.

Unexpectedly, the promoters of seven *U6 small nuclear ribonucleoprotein* genes do not contain USE and TATA elements, which might be Pol II promoters (Kor et al. 2022). The ZmUbi has been successfully used for generating the RNAi plants of *C. richardii* (Plackett et al. 2018), but it requires further investigation on whether it can be used for fern gene editing. According to the transcriptome datasheet of our previous study (Marchant et al. 2022), the transcripts of the putative *CrU6-1*, *CrU6-2*, and *CrU6-3* genes were almost undetectable in the examined tissues (Supplementary Fig. S1), indicating the low expression of *CrU6 s* in *C. richardii*. In most of the CRISPR/Cas9 constructs, the RNA polymerase III-type 3—U3 or U6 promoters are employed for expression of sgRNA in monocots, eudicots, gymnosperms, and bryophytes (Kor et al. 2022). Although we did not use CrU6 promoters in this study, we hypothesize that they can have potential applications in the genome editing of ferns. Moreover, we also obtain the plasmid of pRGEB32-CrU6 (Supplementary Fig. S3E) through replacing the OsU3 promoter of pRGEB32 for future experiments. The U6 promoters from *A. thaliana* showed effective sgRNA driving in other plant species (Gasparis et al. 2018; Hu et al. 2019; Hassan et al. 2021). Therefore, it will be useful to explore the roles of AtU6/AtU3 and CrU6/CrU3 in the gene editing of ferns.

In the future, direct transformation of gametophytes for gene functions in apogamy (Bui et al. 2018) may provide a clue to the evolution of asexual reproduction in land plants, permitting comparison of fern apogamy to somatic embryogenesis and apomixis in angiosperms (Kinosian and Wolf 2022). Despite the great potential, several issues still limit the efficiency of CRISPR/Cas9 as a tool for mitigating plant stresses (Deng et al. 2022). For instance, the inactivation of some genes through gene editing often results in disease resistance, but it is also associated with pleiotropic effects such as inhibition of plant growth, phenotypic abnormalities, and increased susceptibility to abiotic stress and other pathogens (Ma et al. 2018). Abiotic stress tolerance usually depends on complex mechanisms controlled by multiple genes (Adem et al. 2020; Shabala et al. 2020; Tripathi et al. 2020; Wang et al. 2023a, 2023b), implicating the need to develop multiplex CRISPR-based approaches for ferns.

### Advantages of using gametophytes in the transformation of ferns

Bryophytes, ferns and lycophytes rely on free-living gametophytes for reproduction (Fouracre and Harrison 2022). Unlike mosses and liverworts whose dominant generation is the gametophyte (Frangedakis et al. 2023), the dominant generation in ferns is the sporophyte. The spores of ferns are shed by the sporophytes and develop into free-living gametophytes (Bui et al. 2018). This life cycle of ferns provides an opportunity to use gametophytes as targets for transgenesis (Kinosian and Wolf 2022). This is in stark contrast to the transformation protocol for angiosperm species, where the immature embryo, callus, flowers and protoplasts are usually used for efficient stable transformation (Altpeter et al. 2016). Except a 2-month spore dormant period after harvest (Supplementary Fig. S4C), the advantages of using gametophytes are relatively simple and reproducible using large quantity of spores (Bui et al. 2018), which are fast to germinate, easy to manage, and quick to grow on solid medium compared to laborious embryo separation and callus induction needed for genetic transformation of many angiosperms (Ishizaki et al. 2016).

RNAi was made possible through direct uptake of dsRNA into germinating spores of *C. richardii* (Stout et al. 2003) and *Marsilea vestita* (Klink and Wolniak 2001). Particle bombardment of DNA constructs into gametophytes has also been demonstrated in *C. richardii* (Rutherford et al. 2004) and *Adiantum capillus*-*veneris* (Kawai et al. 2003; Kawai-Toyooka et al. 2004) (Table 1). Transgenesis in ferns was demonstrated in *C. thalictroides* and *P. vittata* with spores by *Agrobacterium*-mediated transformation and particle bombardment transformation, respectively (Muthukumar et al. 2013). A tractable particle bombardment transgenesis system using sporophytes has been established in *C. thalictroides* and *C. richardii* (Plackett et al. 2014; Plackett et al. 2015). Callus tissues were induced from young sporophytes, and then bombarded with a GUS reporter and hygromycin selection (Plackett et al. 2014). This method requires callus induction similar to transformation protocols of angiosperms followed by sporophyte regeneration. Here, we optimized the timing of enzyme treatment, OD value of *Agrobacterium*, the suitable concentrations of hygromycin selection, and planting density in Petri dishes, by which we achieved a higher transformation efficiency close to 10% in the overexpression of *CrSAL1* (Table 1, Fig. 2). The high transformation efficiency will enhance our understanding of the function of important genes in the biology, evolutionary, future agricultural, and medicinal applications of ferns.

### Conserved evolution and functional divergence of SAL genes family

Plant *SAL1s* have been extensively reported to be involved in phytohormone signaling (Ishiga et al. 2017) (e.g. ABA, salicylic acid, jasmonic acid, and auxin) and adaptive responses to stresses (Jia et al. 2019) such as *Fusarium graminearum* (Yu et al. 2015), salt (Chen et al. 2011), drought (Abdallah et al. 2022), cold (Shen et al. 2023), high light (Estavillo et al. 2011), oxidative stress (Chan et al. 2016), and cadmium (Xi et al. 2016). Due to its distinct effects on different cellular processes, the underlying molecular mechanisms of *SAL1* in stress responses appear to be complex (Jia et al. 2019). In *A. thaliana*, there are four SALs (AT5G63980, AT5G64000, AT5G63990, AT5G09290) and two homologs [inositol monophosphatase, AT5G54390 (Arabidopsis Halotolerance 2-like, AHL) and AT4G05090] (Shin et al. 2019). AtSAL1 plays a negative role in stress response pathways that are predominantly ABA-dependent and ABA-independent (Wilson et al. 2009). The function of SAL1 was explored through *sal1* mutants in *A. thaliana* (Wilson et al. 2009) and *T. aestivum* (Abdallah et al. 2022), while only a few reports were overexpression *SAL1* in plants (Gasparic et al. 2013; Ku et al. 2013). Interestingly, many *CrSAL1-OE* plants failed to develop normally and complete the life cycle (Supplementary Fig. S5) may be due to the higher cellular ROS accumulation (Fig. 5B) disrupting plant development (Singh et al. 2024).

In this study, *crsal1-2* mutant displayed ABA-sensitive stomatal phenotype, which is in accordance with *fry1* (Xiong et al. 2001) and *sal1*-*8* (Pornsiriwong et al. 2017) that were more sensitive to ABA in *A. thaliana* compared to the WT. In addition, *CrSAL1-OE-1* overexpression plants exhibited a reduced response to ABA-induced stomatal closure, which is in agreement with a previous report that ectopic expression of soybean (*Glycine max*) *GmSAL1* in *A. thaliana* decreased the ABA-induced stomatal closure (Ku et al. 2013). *A. thaliana alx8* also showed low *A* and *g_s_* (Rossel et al. 2005) and *old101* maintained lower ROS levels (Shirzadian-Khorramabad et al. 2022). Interestingly, *crsal1-2* showed significantly less ROS production in the guard cell and decreased photosynthetic parameters (e.g. *A*, *g_s_*, and *VPD*) than WT and *CrSAL1-OE-1* plants (Figs. 4 and 5), indicating the functional similarity of *SAL1s* in the two species.

Our previous study showed that SAL1 and its chloroplast transit peptides were conserved in chlorophyte algae and land plants (Zhao et al. 2019). 197 *SAL* genes in 53 *Chlorophyta* and *Embryophyta* species were identified (Supplementary Fig. S7) through PLAZA platform (Van Bel et al. 2022). The *SAL* gene family was greatly expanded in monocots (e.g. *T. aestivum*, *Phyllostachys edulis*) and eudicots (e.g. *Glycine max*, *Brassica napus*), but not in bryophytes and ferns. Gene expression profiles of *SALs* showed that some of them are specifically expressed in the reproductive organs, leaf, and root (Proost and Mutwil 2018). Interestingly, *AtSAL1* showed high expression in many organs such as root, stem, leaf, flower, seed, reproductive organs, and meristem (Supplementary Table S2). *AtSAL2* was preferentially expressed in the leaf, while *AtSAL4* displayed specific expression in the root, implying their different roles in these tissues. Drought-induced the expression of *Zm00001e039578_P001* (*GRMZM2G152757*, *SAL1*) in maize (Kim et al. 2021), which was also involved in photoperiod at the vegetative-tasseling stage (Wang et al. 2017) and osmotic stress response (Yu et al. 2018). Interestingly, red fluorescence of RFP-SAL (Pp3c3_21240V3.1) was observed in the cytosol of moss *Physcomitrium patens* cells (Cross et al. 2017), implying the diverse biological functions of SALs. Expression analysis demonstrated that some *SAL* genes function in leaf and root of gymnosperms and lycophytes and others are important for the reproductive organs of angiosperms, illustrating that neofunctionalization of *SAL* genes might coincide with the emergence of expansion in angiosperms. However, the study of SALs mainly focused on the *A. thaliana* (Jia et al. 2019). Thus, investigations of the molecular mechanisms of SALs through gene editing are important for enhancing abiotic stress tolerance in crops and addressing key evolutionary biology questions in important early divergent plant lineages such as ferns.

## Materials and methods

### Plant materials and growth conditions

The *Ceratopteris richardii* genotype Hn-n with a fully sequenced and assembled genome (Marchant et al. 2022) was used in our study. Plants were grown in a GEN 1000 (CONVIRON, Manitoba, Canada) at 16 h of light/8 h of dark, 28 °C, 80% relative humidity, and fluence of 100 *μ*mol m^–2^ s^−1^. Gametophytes were grown with 1.5% (w/v) of 1× MS in an agar medium at a pH of 5.9 (Plackett et al. 2015). Spores were sterilized by incubating for 5 min in sodium hypochlorite solution [1% (v/v) chlorine], which was subsequently removed by three sequential rinses with sterile distilled water at 23 °C. Spores were then imbibed in distilled water and incubated for 3 days in darkness before sowing (Plackett et al. 2014; Withers et al. 2023). The spores were imbibed in 1 mL sterile water in the Petri dish, which was sealed with foil and incubated at 28 °C for 7 days and germinated. One-month-old gametophytes can be used for the transformation of *Agrobacterium* (*Agrobacterium tumefaciens*).

### Vector construction and Agrobacterium-mediated transformation of gametophytes

It was reported that Pol III promoters were used to drive sgRNA in plant genome editing (Hassan et al. 2021). Therefore, we used the sequences of the Arabidopsis (*Arabidopsis thaliana*) *U3-b* (X52629, *AT5G53902*), *U6-26 snRNA* (X52528, *AT3G13857*), and the wheat (*Triticum aestivum*)*U6* gene (X63066, *ENSRNA050022746-T1*) (Poovaiah et al. 2021) sequences to compare with the *U6* sequence in *C. richardii* (https://phytozome-next.jgi.doe.gov/info/Crichardii_v2_1). However, we didn’t obtain any orthologous genes of *AtU3* but *AtU6*. Therefore, the upstream *CrU6-2* (*Ceric.13G012300*) promoter regions (Supplementary Fig. S2) were used in the pRGEB32-CrU6 to replace the OsU3. Interestingly, Pol II promoters also were reported to drive sgRNA in rice (*Oryza sativa*)(Tang et al. 2016) and *T. aestivum* (Luo et al. 2021). Thus, we also cloned the *CrActin* promoter (*Ceric.26G012600*) of *C. richardii* through alignment with the *PvActin* of *P. vittate* (Muthukumar et al. 2013). The pRGEB32-CrActin was obtained by replacing the OsU3. Besides, the native ZmUbi promoter in the pRGEB32-OsU3 was replaced by the enhanced 35S promoter (Sugano et al. 2014).

Overexpression and CRISPR/Cas9 constructs were generated utilizing the assembly technology (Bai et al. 2020). Briefly, the PCR products of full-length coding sequences (CDS) were cloned into the vector pJET1.2/blunt using CloneJET PCR Cloning Kit (Thermo Fisher Scientific, Waltham, MA USA) (Awasthi et al. 2021), and then transformed into DH5α competent cells (Life Technologies, Waltham, MA USA). Plasmid purification was performed with a GeneJET Plasmid Miniprep Kit (Thermo Fisher Scientific, Waltham, MA USA) (Lorenzo et al. 2023) and the resulting plasmid DNAs were validated by sequencing. The correct sequence was introduced into the destination vectors pCAMBIA1300-2× 35S (enhanced CaMV 35S promoter) at the restriction enzyme sites *BamHI* and *PstI* (New England BioLabs, Ipswich, MA, USA). The sgRNAs were designed through CRISPR-P 2.0 (http://crispr.hzau.edu.cn/cgi-bin/CRISPR2/SCORE) (Liu et al. 2017a, 2017b, 2017c). To generate CRISPR/Cas9 plasmid, fragments containing tRNA–sgRNA1 fusion and gRNA–tRNA–sgRNA2 fusion were obtained through pGTR (Supplementary Fig. S3B) as a template (Xie et al. 2015; Wang et al. 2018). The PCR products were then cloned into pRGEB32-CrActin vector at the restriction enzyme site *BsaI* (Fu et al. 2022; Kuang et al. 2022). All constructs were introduced into the *Agrobacterium* strain GV3101. Stable genetic transformation of *C. richardii* plants was performed as described previously with modification (Bui et al. 2015, 2017). More details can be found in Supplementary Materials and Methods.

After *Agrobacterium* infection, gametophytes were grown in MS media with 5 mg/L of hygromycin and 100 mg/L of cefuroxime for 30 days. Then, the sporophytes were transferred to new MS media containing 20 mg/L of hygromycin and 100 mg/L of cefuroxime for 30 days. Sporophytes were then transplanted to pots containing a premium potting mix (Scotts Osmocote, Bella Vista, Australia) with the cover to keep high humidity. The plants were watered and fertilized fortnightly with a nutrient solution at 0.5 g/L (Thrive Soluble Fertilizer, Yates, Padstow NSW, Australia). T1 sporophytes grown without hygromycin selection and transgenic individuals were subsequently identified by hygromycin selection on MS media (Plackett et al. 2015).

### Promoter activity assays

The promoters of *CrActin* and *CrU6* were amplified via PCR by the pairs of primers (Supplementary Table S1). Promoters were inserted into pNC-Green-LUC and pNC-121-Pro-GUS vectors by the assembly technology (Yan et al. 2023). All recombinant constructs were introduced into the *Agrobacterium* strain GV3101. And then, they were transformed into the leaves of tobacco (*Nicotiana benthamiana*) for 48 h. The luciferase (LUC) signals were obtained via a Tanon 5200 chemiluminescent imaging system (Jiang et al. 2022). Besides, LUC and REN (renilla luciferases) activities were performed by the Dual Luciferase Reporter Gene Assay Kit (Yeasen). GUS (β-glucuronidase) histochemical staining was conducted using the GUS stain Kit (Coolaber) according to previous protocol (Plackett et al. 2014).

### RT-qPCR analysis of transgenic plants

For expression analysis of *CrSAL1*, total RNA was extracted from infertile leaves through RNeasy Plant Mini Kit (QIAGEN) (Cai et al. 2017, 2021). The cDNA synthesis was performed by QuantiTect Reverse Transcription Kit (QIAGEN) and the synthesized cDNA was diluted 5 times before Quantitative real-time PCR (qPCR) experiments. The qPCR was conducted for three biological replicates using a QuantiNova SYBR Green PCR Kit (QIAGEN) on a LightCycler 96 Real-Time PCR System (CFX Connect) (Jiang et al. 2020). Expression levels were normalized against the *CrACTIN* reference gene (Plackett et al. 2018). The relative expression levels of genes were performed from cycle threshold values by 2^−ΔΔCt^ procedure (Feng et al. 2020; Jiang et al. 2022). All primers were designed using Primer Premier 6.0 (PREMIER Biosoft, San Francisco, CA, USA) or SnapGene Viewer (GSL Biotech LLC, Boston, MA, USA) in this study (Supplementary Table S1).

### Subcellular localization

Subcellular localization of CrSAL1 was performed according to the previous study (Feng et al. 2020). The coding regions of *CrSAL1* were amplified and cloned into pCAMBIA1300-GFP (Fu et al. 2022) by the restriction enzyme site *KpnI* and *XbaI*. The resulting plasmids were transferred into *Agrobacterium* strain GV3101. *Agrobacterium* harboring the vector was grown overnight in Luria-Broth (LB) medium containing 25 mg/L of Rifampin and 50 mg/L of Kanamycin (Jiang et al. 2022). After centrifugation, *Agrobacterium s* was resuspended through the infiltration buffer [10 mm 2-(N-morpholino) ethanesulfonic acid (MES)-KOH (pH 5.7), 10 mm MgCl_2_, 100 μM acetosyringone (AS)] to achieve OD600 = 0.8. The suspension was infiltrated into the abaxial air spaces of 4-week-old *N. benthamiana* leaves using a 1-mL syringe without a needle to transiently express (Feng et al. 2020). GFP fluorescence was detected by using a confocal microscopy (SP5, Leica Microsystems GmbH, Wetzlar, Germany). Confocal imaging settings were excitation at 488 nm, emission at 500 to 530 nm (GFP) and 600 to 660 nm (chloroplast), gain at 750, and intensity at 1% (Deng et al. 2021).

### Measurement of reactive oxygen species (ROS)

The production of ROS in guard cells of *CrSAL1* transgenic and WT plants was detected using a fluorescent indicator 2′,7′-dichlorodihydrofluorescein diacetate (CM-H_2_DCFDA, Life Technologies, Waltham, MA USA) (Cai et al. 2017). Epidermal peels were incubated with an opening buffer [10 mm KCl and 5 mm MES at pH 6.1 with Ca(OH)_2_] for 30 mins in the stomatal assays. The samples were then loading with 20 *µ*M CM-H_2_DCFDA for 30 min in the dark, followed by a 5 min rinse with a measuring buffer [50 mm KCl and 10 mm MES at pH 6.1 with NaOH] to remove excess dye (Cai et al. 2021). The epidermal peels were then incubated in the measuring buffer for confocal microscopy imaging with excitation at 488 nm and emission at 510 to 540 nm (SP5, Leica Microsystems GmbH, Wetzlar, Germany). ROS intensity was calculated using Image J software (NIH, USA).

### Gas exchange measurements

Gas exchange measurements were measured on the *C. richardii* fully expanded infertile leaf by LI-6400 infrared gas analyzer (LI-COR, USA) (Liu et al. 2017a, 2017b, 2017c; Qiu et al. 2023). The parameters are net CO_2_ assimilation (*A*), stomatal conductance (*g_s_*), leaf transpiration rate (*E*), and VPD. Leaf chamber conditions were maintained at a flow rate of 500 mol s^−1^, 70% relative humidity and 400 ppm reference CO_2_. Irradiance levels were set at 0, 20, 50, 100, 200, 300, 500, 800, 1000, and 1500 *μ*mol m^−2^ s^−1^ for light response curve measurement.

### Stomatal assay

The stomatal assay was determined from the abaxial surface of the fully expanded and mature leaves as described in our previous work (O'Carrigan et al. 2014; Liu et al. 2017a, 2017b, 2017c; Plackett et al. 2021). For these measurements, fully expanded infertile leaves were removed from the chamber and placed in Petri dishes on tissue paper soaked in the opening buffer. The lower leaf epidermis was quickly peeled off and placed it on slides with the opening buffer. Stomatal morphology was calculated from the leaf epidermis through a light microscopy and imaging system (Nikon, Tokyo, Japan). Treatment was applied as ABA (50 *μ*M) measured for another 60 min. The pictures were imported into the ImageJ software for the analysis of multiple parameters. Guard cell length, guard cell width, stomatal pore area, and stomatal pore width were recorded (Pan et al. 2022). There were 20 to 30 stomata with three biological replicates for each treatment and genotype.

### Statistical analysis

Data were shown as means with standard errors of three biological replicates. The SPSS 26.0 software (IBM, USA) was employed to perform the analysis of variance (ANOVA) and means were compared by Duncan's multiple range tests.

### Accession numbers

Sequence data from this article can be found in the GenBank data libraries under accession numbers (SAL1, MH686366).

## Supplementary Material

kiae473_Supplementary_Data

## Data Availability

All data are incorporated into the article and its online supplementary material.

## Supplementary data

The following materials are available in the online version of this article.

**Supplementary Figure S1.** Expression and alignment of sequences of U6 small nuclear RNA genes in diverse tissues of *C. richardii*.

**Supplementary Figure S2.** The sequences of the Actin/U6 promoter from *C. richardii* and 2× CaMV 35S promoter, and the activity of CrActin and CrU6 promoters.

**Supplementary Figure S3.** Schematic diagram of the CRISPR/Cas9 gene editing system and plasmid information of CRISPR/Cas9 and overexpression for genetic transformation in *C. richardii*.

**Supplementary Figure S4.** Growth of gametophytes and sporophytes for testing hygromycin sensitivity and germination in *C. richardii*.

**Supplementary Figure S5.** Genotyping and phenotyping of *CrSAL1* overexpression *C. richardii* plants.

**Supplementary Figure S6.** Phenotype of *CrSAL1* and *CrPDS* CRISPR/Cas9 plants and *CrSAL1-OE* plants of *C. richardii* on the hygromycin selection medium.

**Supplementary Figure S7.** Tandem and block gene duplicate of *SAL* genes family in *Chlorophyta* and *Embryophyta*.

**Supplementary Table S1.** List of primer sequences used in this study.

**Supplementary Table S2.** Expression patterns of plant SALs in different tissues and evolutionarily important lineages of plants.

## Dive Curated Terms

The following phenotypic, genotypic, and functional terms are of significance to the work described in this paper:

AT3G13857 Gramene: AT3G13857
AT3G13857 Araport: AT3G13857

AT5G53902 Gramene: AT5G53902
AT5G53902 Araport: AT5G53902

CRU3 Gramene: AT4G28520
CRU3 Araport: AT4G28520

APX2 Gramene: AT3G09640
APX2 Araport: AT3G09640

ALMT1 Gramene: AT1G08430
ALMT1 Araport: AT1G08430

SLAC1 Gramene: AT1G12480
SLAC1 Araport: AT1G12480
