# Supplementary material for: Efficient gene editing of a model fern species through gametophyte-based transformation
Source: Plant Physiol. 2024 Sep 12;196(4):2346–61. doi: 10.1093/plphys/kiae473 (PMC11638000; doi:10.1093/plphys/kiae473)
Supplement: kiae473_Supplementary_Data [file kiae473_supplementary_data.pdf]

Supplementary Data

A

U6 small nuclear ribonucleoprotein	Immature gametophyte	Mature gametophyte	Young sporophyte	Expanding leaf	Development leaf	Fertile leaf	Sterile leaf	Stem	Root	Sori
Ceric.17G074700	2.67	2.72	6.38	26.08	11.97	19.18	14.03	15.17	23.14	44.58
Ceric.33G040100	2.55	1.54	2.58	20.53	7.71	14.57	6.24	11.86	20.44	19.37
Ceric.09G088700	5.80	1.07	1.05	19.94	5.63	6.96	4.35	6.90	11.19	16.84
Ceric.02G026900	2.68	0.74	1.75	14.62	7.43	10.83	9.68	13.45	10.79	30.52
Ceric.1Z290000	3.21	0.00	1.83	18.06	8.41	12.25	10.89	17.56	16.33	33.55
Ceric.03G070800	36.70	5.18	11.77	153.92	39.03	64.34	23.02	51.12	87.96	156.04
Ceric.03G071600	18.61	4.77	5.11	43.90	24.75	44.58	30.27	43.58	53.41	71.81
Ceric.13G012200 (*CrU6-1*)	0.00	0.00	0.00	0.00	0.00	0.00	0.00	0.00	0.00	0.08
Ceric.13G012300 (*CrU6-2*)	0.07	0.00	0.00	0.00	0.00	0.00	0.08	0.00	0.00	0.53
Ceric.1Z176900 (*CrU6-3*)	0.00	0.00	0.00	0.00	0.00	0.00	0.02	0.00	0.00	0.36

B

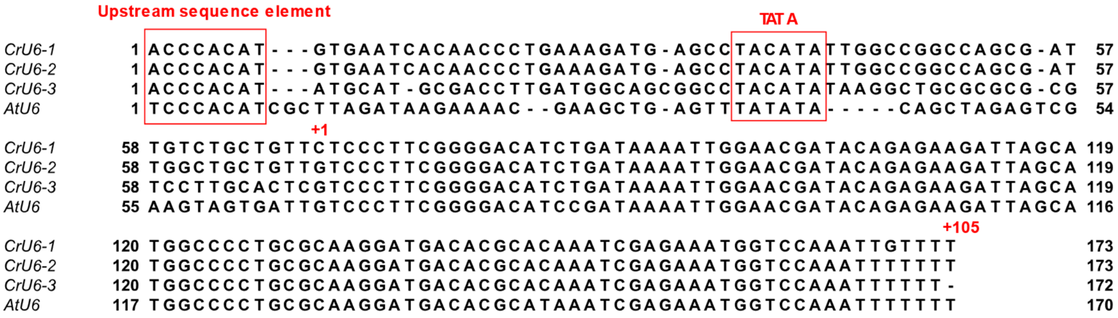

Supplemental Figure S1. Expression and alignment of sequences of *U6* small nuclear RNA genes in diverse tissues of *C. richardii*. (A) Expression of ten *U6* small nuclear RNA genes in diverse tissues. Original data was obtained from our previous study (Marchant et al., 2022). It was showed using the average TPM (transcripts per million) value with three biological replicates. Blue indicating low expression and red indicating high expression. (B) Alignment of the three putative *Ceratopteris richardii* U6 small

nuclear RNA promoter sequences with those from *Arabidopsis thaliana*. *CrU6-1* (*Ceric.13G012200*), *CrU6-2* (*Ceric.13G012300*), and *CrU6-3* (*Ceric.1Z176900*) contain a highly conserved region (+1 to +105). The putative transcription start site of U6-snRNA is shown as +1. Red boxes indicate upstream sequence element (USE) and TATA elements. The 2-kb upstream region of CrU6 might be used to express the guide RNA.

A

Actin promoter of *C. richardii*:

916 bp

CTAGATCTCTTTAATTCTGCCTTTACATACCTTCACTATTTAGCTTAGATTGTG
TTTTCTGTGTTTCGTATAAGGGATCTTTTTTATTTTCGTGCTGCTTAGCGTTC
AACTCAGGTTTTAAATTTGTTTTATCAACTAGGTCCATTATCTAACAAAGCT
AAGGGAAAATGGCTGATGTGGACGAAGTTCAACCGCTTGTTTGTGATAATG
GTTCTGGGATGGTCAAGGTAATGCCAAAGTTTTGTCTGGTTTCTTGTCTATT
GCTTCATGTACAACAACAACACATCCATTTTCTTCAATTCAGGCTGGATTTG
CTGGCGATGATGCCCCTCGGGCTGTATTTCCTAGCATTGTGGGTCGTCCTAG
ACATACTGGGGTCATGGTTGGTATGGGCCAAAAAGATGCATATGTTGGTGAT
GAGGCTCAATCAAAGCGTGGGATTCTTACATTGAAGTACCCTATCGAGCAT
GGTATTGTGACGAATTGGGATGATATGGAGAAGATATGGCACCATACTTTCT
ATAATGAGCTACGTGTTGCTCCAGAGGAGCATCCAGTTCTGCTCACTGAAG
CTCCATTGAATCCGAAGGCCAATCGAGAAAAAATGACACAGATCATGTTTG
ACACGTTCAATGCTCCAGCTATGTATGTTGCAATTCAAGCTGTGTTATCACT
TTATGCAAGTGGAAGGACCACAGGTATGTATTTTATGTCTTTCCTTGAATGC
TATTTGTATTTCAACAAATACATATATAATTTCTAAGTCCAAAAAAATCATGG
GTTGATGAGCTTCTCAGGTATCGTGCTGGATTCTGGTGATGGAGTCACGCA
CACAGTGCCCATTTATGAGGGTTATGCATTGCCCCATGCTATCCTGCGACTG
GATCTTGCAGGTCGGGATTTAACTGATGCTCTT

1600 bp

TGGAGCCCTAAACGAGATACTTAAAACATCATTCACAAAAATTTCAGAGAG
ACACTTTTATTCTCTTATGCATGCTCTACCTTCTCTAACACATAAAATTTCCT
AATTCTTTCATCTTCACATTCAATTTTCAACCAGAAAACATGATGCATATAA
GGTCTAGAGAGAGAATACAAAACTTTTCGTCCTAAAACTTTGCCTACATAC
ATAAAACAACCTGATTGTTCATTCTAAGCATTATCCCCATAGTGTAATTCGGA
AAGTTCAAAATGAATTATTTCACTATGGAAAGGCCTATTACACACATGAACA
TGCATACCGTACATTTATGTATGTCATACACATTATATATTGTTTTCCTTCCTC
TCTAGATATGTACAATATGCATACATACTATATTATCCATGCAATTCCTTTATTT
ATCTCGATGTACTCACTACGACGGTCACACTTGATGCACATTTTGAGGCTCC
TGGTCTATTTTGAGGATCTTGCTCTATCATGTTATGTGGCCAATGTTTCAAGG
AAAACACTCATTGTTAATATCGATTATATTGATGGACTCCCACATTATATACC
AAAAGAAAATTGATCTTTACAATCTCATGCAAGACACACCATTTGGTATTTG
TTAGTAGAAAACATAATTTGAACAAGTAGAGAAATACGAAGGAAAATGATA
AACCTCTTGATTCTCAAGAGAAGGATTGAAATTGAATTGTAGACATGAGAA
CACCGGAGACCGCTAGAGACACTCAAAATTCCATTCTAATTTCATTTCGTTC
TACTTTCATTCCTTCGGTAGATTTGTGTAATTTTTTTCCACTTTCCTTCCTATT
CTTTCGTGCTCAAAATACTTCCATATATCTTTGCTTCACGATTAAAGAAGGT
GAAGACTTCTGATCGTCTCTCATTTTGTCACGATCACGGTAGCAGCCAGCG
TTTGCCGCGCCCATACAGACGCGGACGGAGCAGCCTCACCAACACCAAGG
GGTCTCTGTAAGTCTTTTCTTTCGAGATTTCAGGCCTAAAGTCCATTCATAG
AGATTCGTGAAAATCGAAGCTTTTTCATGCCACTGTTATTGGCTACATATATA
TCAGTCAATGTGGGGGCTGATTGCTCATAACTGGCGCTTGGTTGTTGCATGT

CGGATTCGTAATCCTTAGTTTGCGCAACATGCTTGTAGTGAATGTTCACGAT
CAAGATGTTTTCTGATTCATTGTGGGATCTGCAAGGAACACGTTTAGTTGCG
GCTTCTTCGGCGTTTTTGTGTGCGTGCAATTTCTCTACACAACCTCTCTGAA
AGTGTTTGATAGGACTCCCGCATTTTGTAAATTCTCATTTAAATGGCATTTCT
AGGTTCTAATTGGTCTCATATTGCTGCTTATTCTATAAAATAAATGAAAATTG
TGTAGAAATCTGCTAATATGGTCTAGATCTCTTTAATTCTGCCTTTACATACC
TTCACTATTTAGCTTAGATTGTGTTTTCTGTGTTTCGTATAAGGGATCTTTTTT
ATTTTCGTGCTGCTTAGCGTTCAACTCAGGTTTTAAATTTGTTTTATCAACTA
GGTCCATTATCTAACAAAGCTAAGGGAAA

B
2× CaMV 35S promoter:
GGAGAGGCGGTTTGCGTATTGGCTAGAGCAGCTTGCCAACATGGTGGAGC
ACGACACTCTCGTCTACTCCAAGAATATCAAAGATACAGTCTCAGAAGACC
AAAGGGCTATTGAGACTTTTCAACAAAGGGTAATATCGGGAAACCTCCTCG
GATTCCATTGCCCAGCTATCTGTCACTTCATCAAAAGGACAGTAGAAAAGG
AAGGTGGCACCTACAAATGCCATCATTGCGATAAAGGAAAGGCTATCGTTC
AAGATGCCTCTGCCGACAGTGGTCCCAAAGATGGACCCCCACCCACGAGG
AGCATCGTGGAAAAAGAAGACGTTCCAACCACGTCTTCAAAGCAAGTGGA
TTGATGTGAACATGGTGGAGCACGACACTCTCGTCTACTCCAAGAATATCA
AAGATACAGTCTCAGAAGACCAAAGGGCTATTGAGACTTTTCAACAAAGG
GTAATATCGGGAAACCTCCTCGGATTCCATTGCCCAGCTATCTGTCACTTCA
TCAAAAGGACAGTAGAAAAGGAAGGTGGCACCTACAAATGCCATCATTGC
GATAAAGGAAAGGCTATCGTTCAAGATGCCTCTGCCGACAGTGGTCCCAAA
GATGGACCCCCACCCACGAGGAGCATCGTGGAAAAAGAAGACGTTCCAAC
CACGTCTTCAAAGCAAGTGGATTGATGTGATATCTCCACTGACGTAAGGGA
TGACGCACAATCCCACTATCCTTCGCAAGACCCTTCCTCTATATAAGGAAGT
TCATTTCATTTGGAGAGGACACGCTGAAATCACCAGTCTCTCTCTACAAATC
TATCTCTCTCGAGCTTTCGCAGATCCGGGGGGCAATGAGAT

C
CrU6 promoter of *C. richardii*:
CrU6-1: 516 bp
TTAGGGTTATAGGTGTATGTATATACGTGCGGAGCGGACAAAAAGAGCTCCT
AAAGAGGGAAGAACGTGAGGGGGTGGTGCTCAGTGTTGAACTTTCTATGA
ATGTAGTTTGTTGTAAAGATGCTTTCTAGAATTGGAATTGGAGCTTTTTCTA
GGATTCGAGGGTTTGTGGAATTGGAATTGGAGCTATTTTTTAAGATATAATC
TTGTTTCTGGGGCATTTGACTCCCATAGCTCCATATGTTTTGAGGTTCTCGG
TGCGTTTCCCTCTTCGTGATGGTGTTTGTCGTACTACCTTTGATCTCACTGTT
TTGTTCGTCCGTGTATTGCAGAGGGGTTTTGTGCCGATTGAAAAGCTGTGG
CATTTAGTGTGTTGGTGTGTCGACTGAGGGTAGTTCAACGGAAGAGGCGC
GCGTGGATAGGAATTATTAATAATGTGGGGAGGAGAAAACCCACATGTGAA
TCACAACCCTGAAAGATGAGCCTACATATTGGCCGGCCAGCGATTGTCTGC
T

1000 bp
TGACAACCAACATAGGGGTTACATCATTTCTTTCAACCACGGCTAGTAATTA
AACATTCTAATGCGGAAAATCGAGTGTATCTCAAAAAGTCGAACATAAATA
CCCAAATAATCTCTGCCAAAAGTGTAGCAGACTGAAAACTCTATATGTGCTT
GGCAAAACATTGTCTCAACAATCATTGTGAGTGCACGTGCATTTGGGGAGC
CTAAACCAAGATCTAATAAAAAATTTGATGCTAGTGAGCAAAGCTCTTTTG
GACTTTTAAGTGTTCCGAGTATGCTCATCAACTTTCATGCATTTGAGATACT
CTGATGAGAGGGGTGTCAGCGACGCCCTAGAGGAAGCAGTGACCTTCTCT
GATACCAATTGTTGGACTTCCAGGTCTACGGAAGCACAGGAGTCGCGGAA
GGAAGAGGAAGAAGCTTGGAGAACAAAAGAAGAGTGAATGTGTTACCAC
ATGACTCTAGAAAAACAAGTGATCTATTAGGGTTATAGGTGTATGTATATAC
GTGCGGAGCGGACAAAAAGAGCTCCTAAAGAGGGAAGAACGTGAGGGGG
TGGTGCTCAGTGTTGAACTTTCTATGAATGTAGTTTGTTGTAAAGATGCTTT
CTAGAATTGGAATTGGAGCTTTTTCTAGGATTCGAGGGTTTGTGGAATTGG
AATTGGAGCTATTTTTTAAGATATAATCTTGTTTCTGGGGCATTTGACTCCCA
TAGCTCCATATGTTTTGAGGTTCTCGGTGCGTTTCCCTCTTCGTGATGGTGT
TTGTCGTACTACCTTTGATCTCACTGTTTTGTTCGTCCGTGTATTGCAGAGG
GGTTTTGTGCCGATTGAAAAGCTGTGGCATTTTAGTGTGTTGGTGTGTCGA
CTGAGGGTAGTTCAACGGAAGAGGCGCGCGTGGATAGGAATTATTAATAAT
GTGGGGAGGAGAAAACCCACATGTGAATCACAACCCTGAAAGATGAGCCT
ACATATTGGCCGGCCAGCGATTGTCTGCT

CrU6-2: 516 bp
TTAGGGTTATAGGTGTATGTATATACGTGCGGAGCGGACAAAAAGAGCTCCT
AAAGAGGGAAGAACGTGAGGGGGTGGTGCTCAGTGTTGAACTTTCTATGA
ATGTAGTTTGTTGTAAAGATGCTTTCTAGAATTGGAATTGGAGCTTTTTCTA
GGATTCGAGGGTTTGTGGAATTGGAATTGGAGCTATTTTTTAAGATATAATC
TTGTTTCTGGGGCATTTGACTCCCATAGCTCCATATGTTTTGAGGTTCTCGG
TGCGTTTCCCTCTTCGTGATGGTGTTTGTCGTACTACCTTTGATCTCACTGTT
TTGTTCGTCCGTGTATTGCAGAGGGGTTTTGTGCCGATTGAAAAGCTGTGG
CATTTTAGTGTGTTGGTGTGTCGACTGAGGGTAGTTCAACGGAAGTGGCGC
GCGTGGATAGGAATTATTAATAATGTGGGGAGGAGAAAACCCACATGTGAA
TCACAACCCTGAAAGATGAGCCTACATATTGGCCGGCCAGCGATTGGCTGC
T
1000 bp
TGACAACCAACATAGGGGTTACATCATTTCTTTCAACCACGGCTAGTAATTA
AACATTCTAATGCGGAAAATCGAGTGTATCTCAAAAAGTCGAACATAAATA
CCCAAATAATCTCTGCCAAAAGTGTAGCAGACTGAAAACTCTATATGTGCTT
GGCAAAACATTGTCTCAACAATCATTGTGAGTGCACGTGCATTTGGGGAGC
CTAAACCAAGATCTAATAAAAAATTTGATGCTAGTGAGCAAAGCTCTTTTG
GACTTTTAAGTGTTCCGAGTATGCTCATCAACTTTCATGCATTTGAGATACTT
TGATGAGAGGGGTGTCAGCGACGCCCTAGAGGAAGCAGTGACCTTCTCTG
ATACCAATTGTTGGACTTCCAGGTCTACGGAAGCACAGGAGTCGCGGAAG
GAAGAGGAAGAAGCTTGGAGAACAAAAGAAGAGTGAATGTGTTACCACA

TGACTCTAGAAAAACAAGTGATCTATTAGGGTTATAGGTGTATGTATATACG
TGCGGAGCGGACAAAAAGAGCTCCTAAAGAGGGAAGAACGTGAGGGGGT
GGTGCTCAGTGTTGAACTTTCTATGAATGTAGTTTGTTGTAAAGATGCTTTC
TAGAATTGGAATTGGAGCTTTTTCTAGGATTCGAGGGTTTGTGGAATTGGA
ATTGGAGCTATTTTTTAAGATATAATCTTGTTTCTGGGGCATTTGACTCCCAT
AGCTCCATATGTTTTGAGGTTCTCGGTGCGTTTCCCTCTTCGTGATGGTGTT
TGTCGTACTACCTTTGATCTCACTGTTTTGTTCGTCCGTGTATTGCAGAGGG
GTTTTGTGCCGATTGAAAAGCTGTGGCATTTTAGTGTGTTGGTGTGTCGACT
GAGGGTAGTTCAACGGAAGTGGCGCGCGTGGATAGGAATTATTAATAATGT
GGGGAGGAGAAAACCCACATGTGAATCACAACCCTGAAAGATGAGCCTAC
ATATTGGCCGGCCAGCGATTGGCTGCT

CrU6-3-1: 441 bp
AAGGTGTTGAGCCAAGTGACCAGCTAGGAGTCTCGTCAACACATGGGTGG
GGGGACTCTTGAGAGCCAAGTGGGGCATATTACTTTGATGATGGTTTCTTAT
TATTTGAGAGAGTGGGTGCTTATTAGTTTCATGAGGAATGTTTGAGCCTTAG
ATCGTGGAGTGAGTGAATCTCCATATATTCACGTCTCATTCCCTTTGCGCAA
ATATCAAAACATTTGATGATAGTAGAGTGAAGGAAAGGATTTAAGAGGACC
ACGCGTCCGAAGTAAAAGACAAAAATAAAAAAGGGGCACATGAATGCAAC
TTAATCTCAGTCGCTTGGTAATTTTAGTGCCCACAAAAGTTTGCGTCGCGGG
GTTTTTAAGGCCACAAAACCCACATATGCATGCGACCTTGATGGCAGCGGC
CTACATATAAGGCTGCGCGCGCGTCCTTGCA
1020 bp
ATCCATCTGCCCATATGTCGATATGTTTTTCGCCTTCTTTCTGCAATCCATCC
ACTCTAGAATGTTGAAGAGCCCTTCGGCATACAATTCTTGTCGTTGCATACT
CATGACTAACTGCAATGTTGATATGGAACTTATCACAATGATTTTGGCTAGT
TGAAAGCTAACTGAGAGTGTTTGGGTTGAGGTTCTCCATGGTCTACTATGA
AGAGTGACGCGAAAGTGTCTAAAATGTCATGAAAGAGAGAAACAAGCCTG
CTATGGAAAGTAGAGTAAAAAAAGTAAACGTCATAAGCACACATTATATAC
GTCCATGAACAAGAGGAAAATTGAAATTTTCATTAACGCCCGATAAGCACA
AACTTCGAGGCATAAGATATTACACATACTTTTCATGATTATCACACGAGTTT
CTCTCTCTTCAAAGTTTGCATCAGATACATTTTAAATGCGCTTACACATAATT
TTTTAAAATGATTTGACAACTTTAATCCAGATTAAGCACAACTAAATGAAAT
CCTTAGATGGCCCTTTTTTGGACGTTTTCTGCCCTAATGACAAGGTAACGCT
CTACCTTCGAAGGTGTTGAGCCAAGTGACCAGCTAGGAGTCTCGTCAACA
CATGGGTGGGGGGACTCTTGAGAGCCAAGTGGGGCATATTACTTTGATGAT
GGTTTCTTATTATTTGAGAGAGTGGGTGCTTATTAGTTTCATGAGGAATGTTT
GAGCCTTAGATCGTGGAGTGAGTGAATCTCCATATATTCACGTCTCATTCCC
TTTGCGCAAATATCAAAACATTTGATGATAGTAGAGTGAAGGAAAGGATTT
AAGAGGACCACGCGTCCGAAGTAAAAGACAAAAATAAAAAAGGGGCACA
TGAATGCAACTTAATCTCAGTCGCTTGGTAATTTTAGTGCCCACAAAAGTTT
GCGTCGCGGGGTTTTTAAGGCCACAAAACCCACATATGCATGCGACCTTGA
TGGCAGCGGCCTACATATAAGGCTGCGCGCGCGTCCTTGCA
CrU6-3-2: 1022 bp

CTTCCTGCACAAAGTGTGATGGAGGTGGCATCGAATTGGAGCATAAAGTGA
TCCAACTCTGCATGGAATGGATGATTGGCATTCTTTGTGCAATGGAGGTGG
CATCGAATTGGAGCATAAAGTGATCCAACTCTGCATGGAATGGATGATTGG
CATTGACTTCATGCATAAAGCGATGCCTCCATGGCACACATACTTGGTTCTC
ATCCTAGCATACATACTTCGACTCGGAGCTCGCCAGTTCCCCTCAAAATCCA
TGAAGCCTAGGCATGAGGGTGATCACCTTCTCTGGAGGTTCATCTTCTCCAT
ATCACAGCACAAGGCATGTGGGTCTCATATGGAGTCGGAAGAGCCCTTGTA
AAGGCATTCCAGGATGCCTTCTCAGTATTGGTGAAGGTGTATTTTCGACCAT
TGAAGCAGCTCCCGAGAGAGGTGTAGTCAAGTGGCATAGATGTTAGATTGA
GAGCTACCACAAGGCAAGGCTCTACCTTCGGATTCCCATGGCATATCCTCC
ACTTTTCTTGTGGTGGGAAGACCGCCAAACGGGAATCTATGGGCCATAGCC
ACAAACGAACCGCTCAAAGGTGTTGAGCCAAGTGACCAGCTAGGAGTCTC
GTCAACACATGGGTGGGGGGACTCTTGAGAGCCAAGTGGGGCATATTACTT
TGATGATGGTTTCTTATTATTTGAGAGAGTGGGTGCTTATTAGTTTCATGAGG
AATGTTTGAGCCTTAGATCGTGGAGTGAGTGAATCTCCATATATTCACGTCT
CATTCCCTTTGCGCAAATATCAAAACATTTGATGATAGTAGAGTGAAGGAA
AGGATTTAAGAGGACCACGCGTCCGAAGTAAAAGACAAAAATAAAAAAGG
GGCACATGAATGCAACTTAATCTCAGTCGCTTGGTAATTTTAGTGCCCACAA
AAGTTTGCGTCGCGGGGTTTTTAAGGCCACAAAACCCACATATGCATGCGA
CCTTGATGGCAGCGGCCTACATATAAGGCTGCGCGCGCGTCCTTGCA

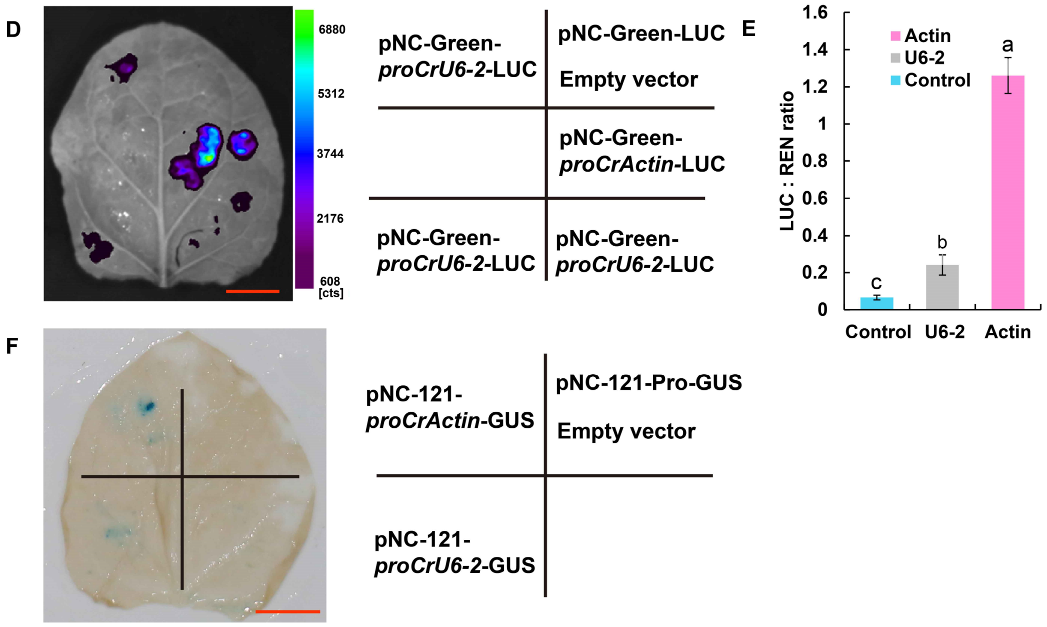

Supplemental Figure S2. The sequences and the activities of the promoters employed in this study. The sequences of the *Cractin* (A), 2× CaMV 35S promoter (B), and CrU6 promoters (C) were listed. The activity of *CrActin* and *CrU6-2* promoters were compared in tobacco leaves transiently expressed LUC (D), dual-luciferase (E) and GUS (F), bar=1 cm. Green indicating low expression and purple indicating high expression. Different letters indicate significant differences compared with the control (empty vector) plants ($P < 0.05$). Values are means of three biological replicates ± SE. The SPSS 26.0 software (IBM, USA) was employed to perform the analysis of variance (ANOVA) and means were compared by Duncan's multiple range tests.

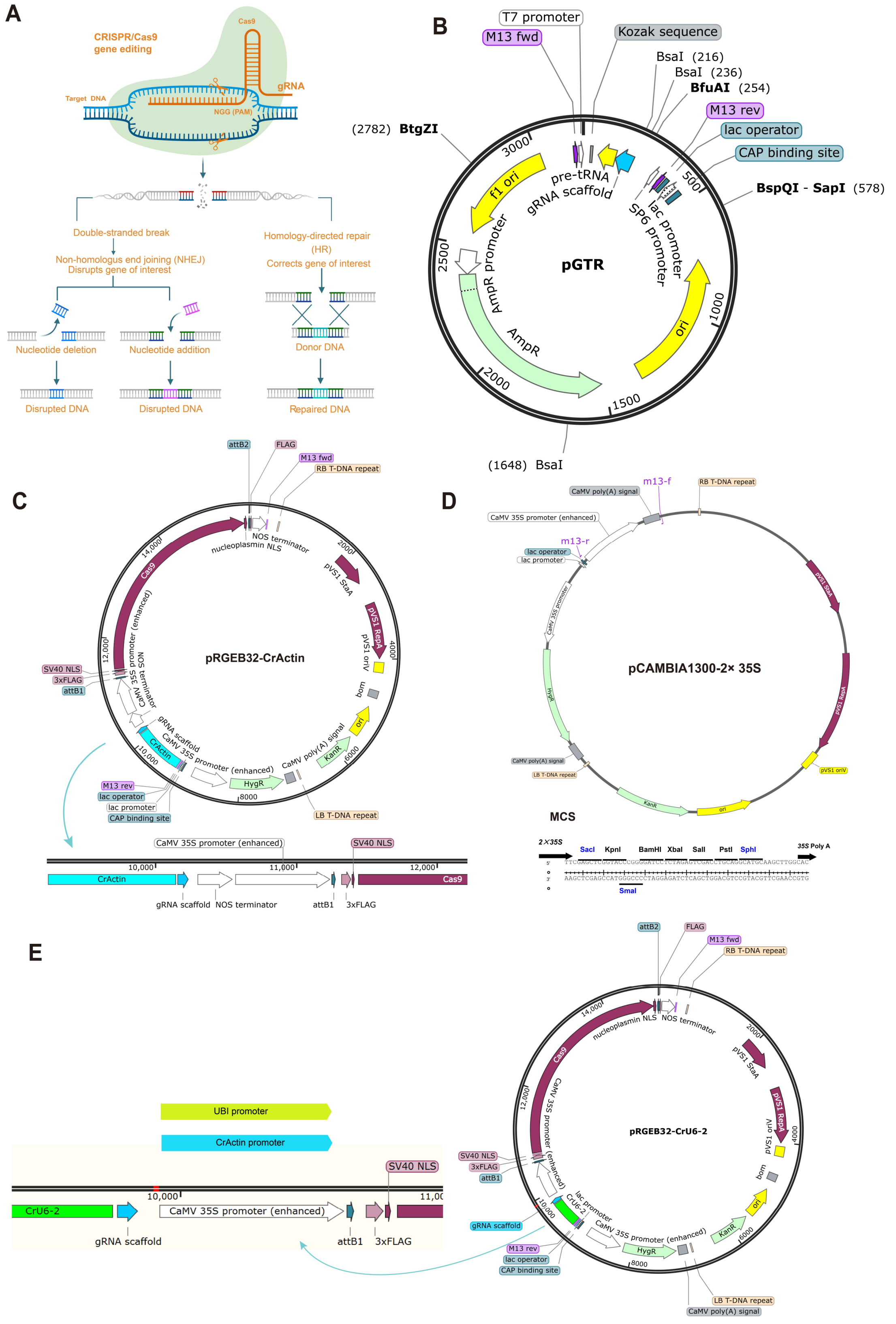

Supplemental Figure S3. Schematic diagram of the CRISPR/Cas9 gene editing system and plasmid information of CRISPR/Cas9 and overexpression for genetic transformation in *C. richardii*. (A) Schematic diagram of CRISPR/Cas9 system. This

figure was drawn through BioRender (https://app.biorender.com/). When Cas9 is complexed with gRNA, it forms a sequence-specific endonuclease. A target-specific DNA sequence of 23 bp is recognized by the Cas9-gRNA complex. The sequence is terminated by an arbitrary nucleic acid and two guanines (NGG, PAM sequence). The 20 bp region can be designed and is known as the target sequence. (B) The map of pGTR plasmid, which was employed as the template for obtaining the polycistron consisting of varied tRNA (sgRNA-tRNA)^n fusion targeting to multiplex editing. The constructs of the modified CRISPR/Cas9 plasmid, pRGEB32-CrActin (C) and pCAMBIA1300-2X35S (D) for gene editing and overexpression in *C. richardii*, respectively. The putative promoter fragment of *CrActin* with the length of916 bp was instead of the *OsU3* promoter, and the native maize ubiquitin promoter (ZmUbi) promoter in the original construct pRGEB32 was replaced by the enhanced 35S promoter, which was designated as pRGEB32-CrActin. (E) The potential CRISPR/Cas9 plasmid (pRGEB32-CrU6-2) of fern, which used CrU6-2 to drive gRNA and enhanced 35S/CrActin/UBI promoter to drive Cas9 protein.

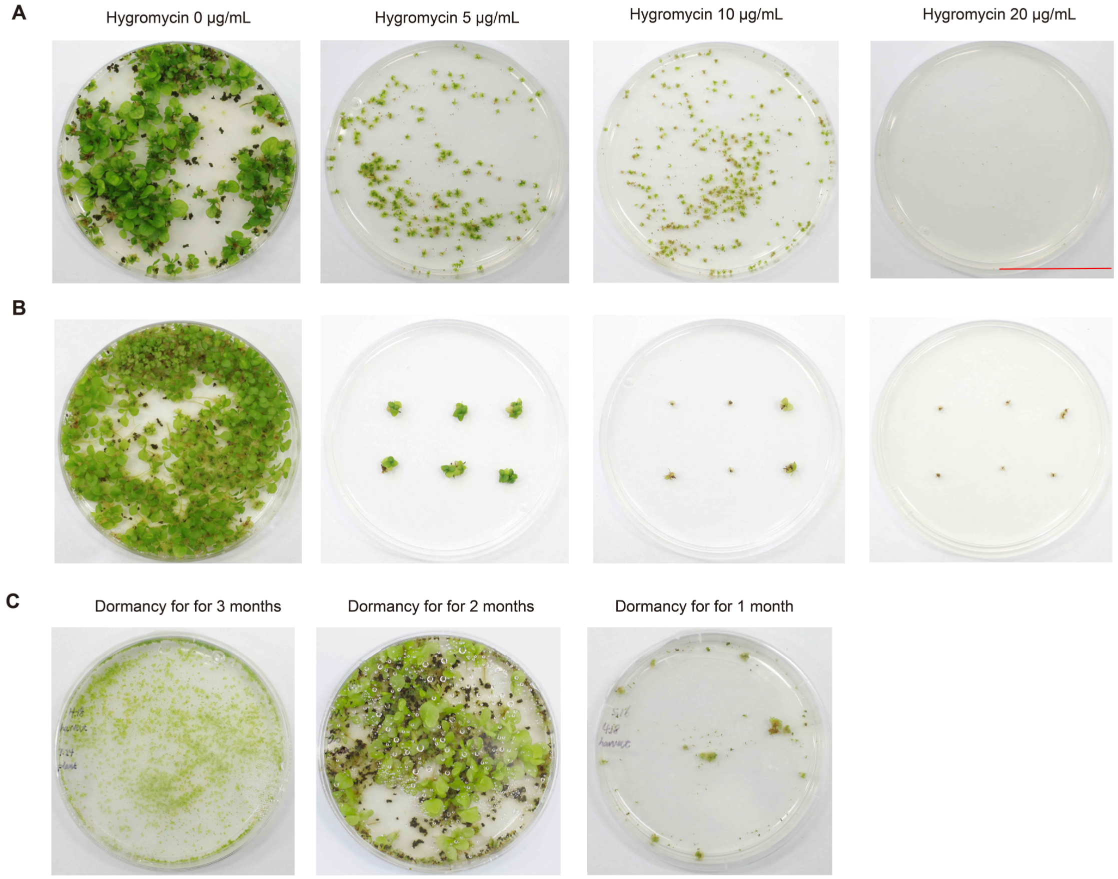

Supplemental Figure S4. Growth of gametophytes and sporophytes for testing hygromycin sensitivity and germination in *C. richardii*. Hygromycin sensitivity of *C. richardii* gametophytes (A) and sporophytes (B). (C) Germination test after dormancy for 1, 2, and 3 months. Bar=5 cm. The scale bar in A refer to all other images in this panel.

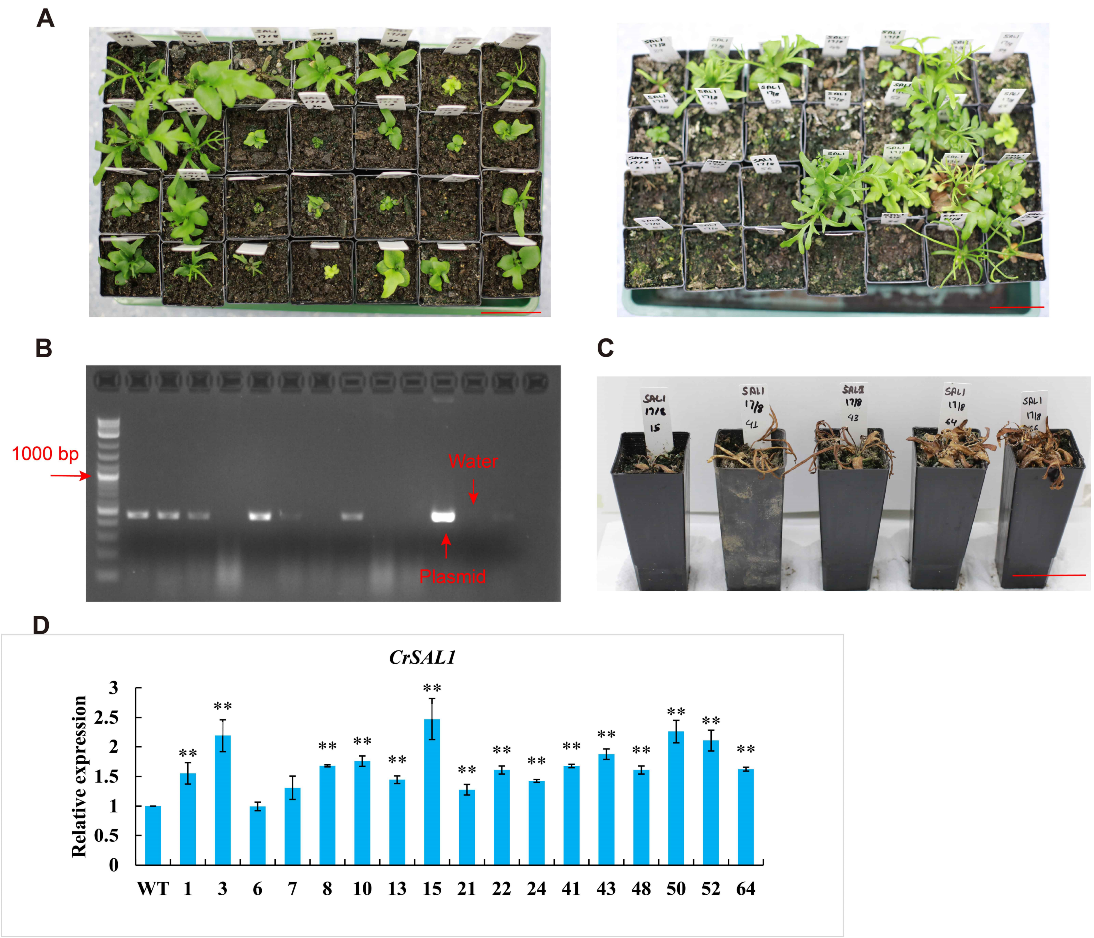

Supplemental Figure S5. Genotyping and phenotyping of *CrSAL1* overexpression *C. richardii* plants. (A) Phenotype photos of *CrSAL1* overexpression plants, bar=5 cm. (B) Identification of transgenic plants using hygromycin primers. The PCR product was 456 bp. (C) Phenotype photos of positive plants, and most transgenic plants were died. (D) Relative expression levels of *SAL* gene in different *CrSAL1* overexpression plants. Asterisks indicate significant differences compared with the WT plants (***P* < 0.01). Values are means of three biological replicates ± SE. The SPSS 26.0 software (IBM, USA) was employed to perform the analysis of variance (ANOVA) and means were compared by Duncan's multiple range tests. The image in Figure S5B appears to be the same image as shown in Figure 2B.

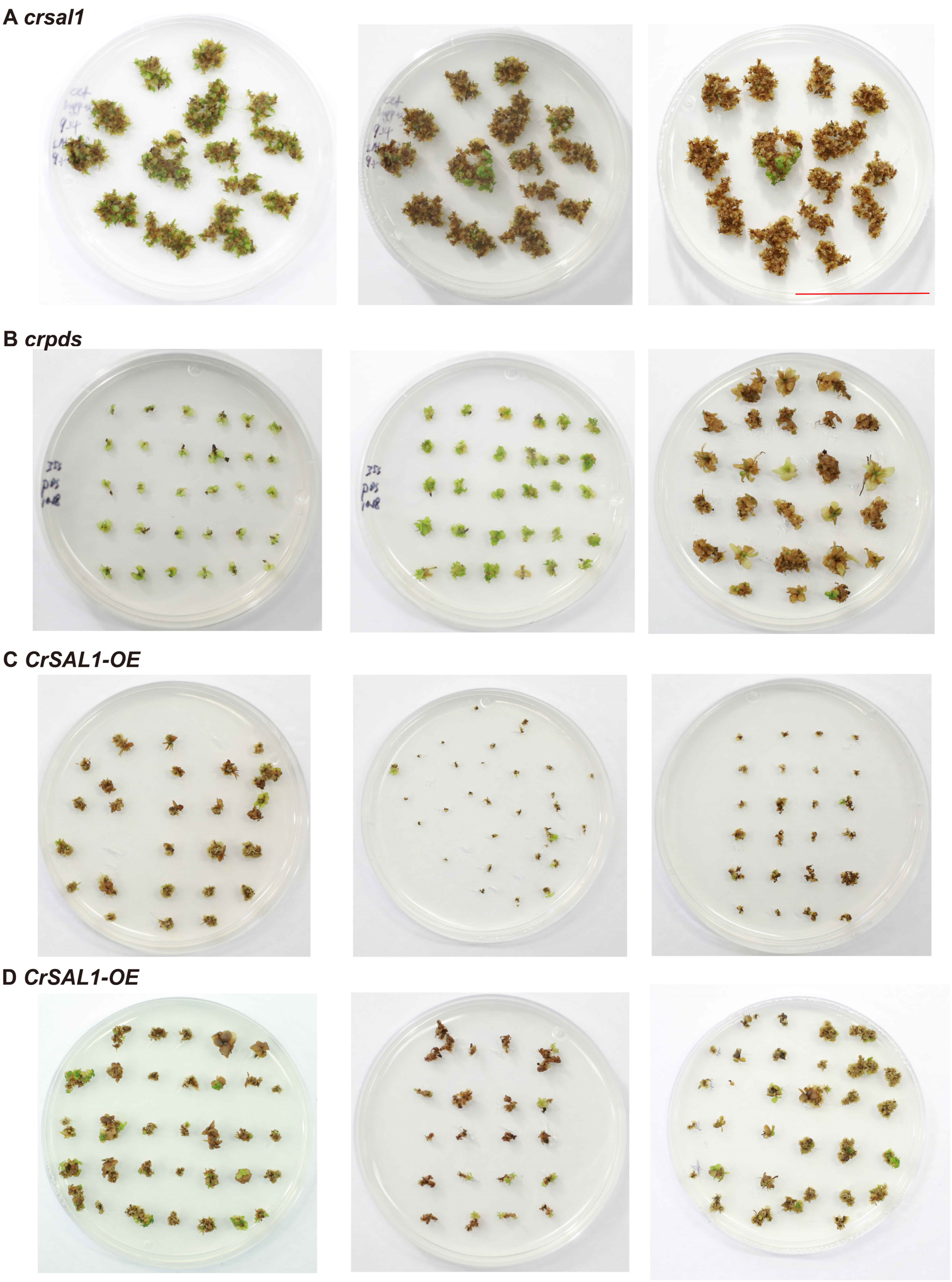

Supplemental Figure S6. Phenotype of *CrSAL1* and *CrPDS* CRIPSR/Cas9 plants and *CrSAL1-OE* plants of *C. richardii* on the hygromycin selection medium. Photos of *CrSAL1* (A) and *CrPDS* (B) CRIPSR/Cas9 plants of *C. richardii* on the hygromycin selection. The images have been reused for the hygromycin treatment of 30 (middle) and 60 (right) days. *CrSAL1-OE* plants of *C. richardii* on the hygromycin selection with 15 min (C) and 2 h (D) treatment of 1.5% (w/v) cellulase before *Agrobacterium* co-

incubation. Bar=5 cm. The scale bar in A refer to all other images in this panel.

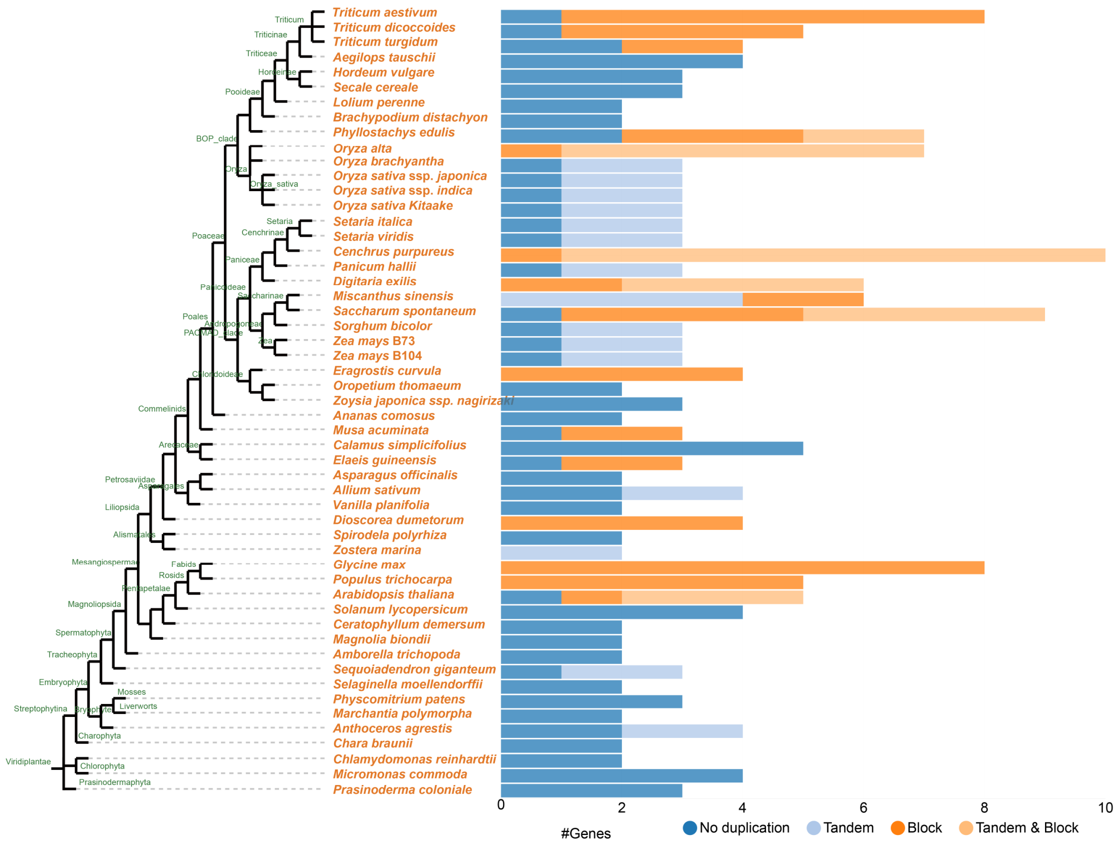

Supplemental Figure S7. Tandem and block gene duplicate of *SAL* genes family in *Chlorophyta* and *Embryophyta*. All the genes number were download from the PLAZA database (https://bioinformatics.psb.ugent.be/plaza/) containing >100 plant and algal species. The phylogenetic tree of distinct species was obtained through TimeTree (http://www.timetree.org/).

Supplemental Table S1. List of primer sequences used in this study.

Purpose	Primer name	Primer Sequence
hygromycin	HPT-F1	GAGCCTGACCTATTGCATCTCC
	HPT-R1	GGCCTCCAGAAGAAGATGTTGG
primer for sequence	35S-F	gacgcacaatcccactatcc
	Nos R	accggcaacaggattcaatc
	pJET1.2-F	CGACTCACTATAGGGAGAGCGGC
	pJET1.2-R	AAGAACATCGATTTTCCATGGCAG
	Cas9-F-jd	GCTTAGCGTTCAACTCAGG
	Cas9-R-jd	GCTCTAAAACAAAAAAGCACCGAC
	M13-F	GTAAAACGACGGCCAGT
	M13-R	CAGGAAACAGCTATGAC
primer for cas9 plasmid	Actin-Pro-F	acatgattacgccaagcttCTAGATCTCTTTAATTCTGCC
	Actin-Pro-R	ctatttctagctctaaaaccgagacctcggtctccAAGAGCATCAGTTAAATCCC
	U6-2-Pro-F	acatgattacgccaagcttTTAGGGTTATAGGTGTATGTATATA
	U6-2-Pro-R	ctatttctagctctaaaaccgagacctcggtctccAGCAGCCAATCGCTGGCC
	Nos_F	GATCGTTCAAACATTTGGCAATAAA
	Nos_R	GATCTAGTAACATAGATGACACCGC
	35s_F	GGAGAGGCGGTTTGCGTATT
	35s_R	ATCTCATTGCCCCCCGGAT
	Geb_nos+35s_f	gcgtgcatgcctgcaggGATCGTTCAAACATTTGGCAATAAA
	Geb_ nos+35s _r	agaattggttctatctccttcgaaATCTCATTGCCCCCCGGAT
Overexpression	OE_SAL1_FM	ttcgagctcggtacccggggatccATGGAGTGCGTGAGAGCG
	OE_SAL1_RM	gtgccaagcttgcatgcctgcagTCATTTATAAACCTTGAGTGCTGC
	OE_CRY4_FM	ttcgagctcggtacccggggatccATGGAGAAGGTTTGTACAGTAG
	OE_CRY4_RM	gtgccaagcttgcatgcctgcagCTATGCTTTTGCTCGAGGTTTTG
	OE_GRF8_f	ttcgagctcggtacccggggatccATGAGCACGGAGAAGGAGCG
	OE_GRF8_r	gtgccaagcttgcatgcctgcagTCACTCTGCATTCTCCAACTTTC
	OE-YSL-F	ttcgagctcggtacccggggatccATGGGTCGAGACGAACAATC
	OE-YSL-R	gtgccaagcttgcatgcctgcagCTATGTAGATGAAAATGACATACAT
Cas9	F1-Cas9-NEW	GGGATTTAACTGATGCTCTTaacaaagcaccagtggtctag
	SAL1-R1	TTTGTACCATCGATAGGATCtgcaccagccgggaatcgaac
	SAL1-F2	GATCCTATCGATGGTACAAAgttttagagctagaaatag
	SAL1-R2	gctatttctagctctaaaacTCATCTAGAAGGGCTAGAGCtgcaccagccgggaatcg
	PDS-R1	AATTATATTGAAGCAGCAGCtgcaccagccgggaatcgaac
	PDS-F2	GCTGCTGCTTCAATATAATTgttttagagctagaaatag
	PDS-R2	gctatttctagctctaaaacTCCTTTTCCTGTAAAATAGTtgcaccagccgggaatcg
	CrYSL-r1	TTGCGTCGATCGCTGTTTCAtgcaccagccgggaatcgaac
	CrYSL-f2	TGAAACAGCGATCGACGCAAgttttagagctagaaatag
	CrYSL-r2	gctatttctagctctaaaacCCGTGAGATTGAGCTTATGCtgcaccagccgggaatcg

	CRY4-R1	TTCGAACCTGAATCTCCGCTtgcaccagccgggaatcgaac
	CRY4-F2	AGCGGAGATTCAGGTTCGAAgttttagagctagaaatag
	CRY4-R2	gctatttctagctctaaaacTCAGGTAAGCTTCCAGAAATtgcaccagccgggaatcg
for cas9 targt sequence	SAL1-1F-Cas9-j	GATGAGGGTCTTGAAAGCTC
	SAL1-1R-Cas9-j	GCATCATTGTCCCTCCACCTC
	PDS-1F-Cas9-j	CTTATGCATGGACCGCCTG
	PDS-1R-Cas9-j	GAGGGATAGGAACAGCCAG
	PDS-2F-Cas9-j	TTGCCTATTTGGATTCACGTG
	PDS-2R-Cas9-j	GCATTGCAGACACATACAGATC
qPCR	SAL1-RT-F-1	TGATGGCTTGGACATGCTGT
	SAL1-RT-R-1	ACCACCCTGAGATCTACCCC
Promoter activity	nc-Actin-Pro-F	cagtggtctctgtccagtcctCCTCTTGATTCTCAAGAGAAGGA
	nc-Actin-Pro-R	cggtctcagcagaccacaagtTTTCCCTTAGCTTTGTTAGATAATGG
	nc-U6-2-Pro-F	cagtggtctctgtccagtcctTTAGGGTTATAGGTGTATGTATATA
	nc-U6-2-Pro-R	cggtctcagcagaccacaagtAGCAGCCAATCGCTGGCC
GFP	GFP_SAL1_FM	ggggacgagctcggtacccggggatccATGGAGTGCGTGAGAGCG
	GFP_SAL1_RM	cccttgctcaccatgtcgactctagaTTTATAAACCTTGAGTGCTGC

Supplemental Table S2. Expression patterns of plant SALs in different tissues and evolutionarily important lineages.

Gene	Root	Flower	Leaf	Stem	Female	Seeds	Male	Apical meristem	Root meristem
AtSAL1	81.08	50.5	32.87	75.99	35.81	75.14	76.26	69.67	59.41
AtSAL2	9.42	3.78	30.61	5.53	10	2.43	2.78	1.29	4.93
AtSAL3	44.79	27.55	3.03	22.84	19.54	31.82	60.6	8.71	12.57
AtSAL4	15.14	0.55	3.36	3.33	9.05	0.54	2.86	0.23	6.8
AtAHL	53.6	76.85	51.51	198.6	72.4	82.63	73.46	90.06	190.59
Solyc02g078860.3.1	11.83	12.56	13.96	21.91	6.69	9.47	71.29	12.96	0.77
Solyc05g056490.4.1	42.37	28.72	18.18	26.81	23.68	46.78	31.11	37.5	58.29
Solyc12g013560.2.1	3.46	6.47	2.56	4.37	4.79	4.52	13.51	8.07	6.51
Zm00001e023656_P002	13.22	8.88	6.5	5.27	5.03	5.2	1.29	6.76	3.3
Zm00001e039577_P002	57.41	62.37	61.12	53.88	36.67	50.99	15.43	51.41	89.63
Zm00001e039578_P001	21.66	50.14	71.95	34.67	49.99	25.07	6.46	23.13	18.45
LOC_Os02g56170.1	20.43	22.26	42.35	14.81	8.72	4.07	1.05	8.32	4.1
LOC_Os12g08270.1	47.92	1.94	3.22	2.51	1.41	1.18	0.22	1.22	8.04
LOC_Os12g08280.1	59.69	38.52	47.48	63.19	22.62	18.94	5.45	34.62	27.97
AMTR_s00003p00261960	13.49	29.86	15.31	-	47.13	-	13.93	17.08	-
AMTR_s00017p00255870	26.91	44.94	20.81	-	46.25	-	15.24	61.75	-
MA_10427304g0020	-	5.94	13.03	6.54	-	-	-	-	-
MA_10433905g0010	-	15.85	40.02	96.14	-	-	-	-	-
MA_10434544g0010	-	19.08	23.34	28.46	-	-	-	-	-
MA_10514g0020	-	0	0.05	0.12	-	-	-	-	-
MA_121730g0010	-	0	0	0	-	-	-	-	-
MA_348554g0010	-	0	0.84	1.21	-	-	-	-	-
MA_417053g0010	-	0	0	0.08	-	-	-	-	-
MA_601652g0010	-	0	0.3	0.08	-	-	-	-	-
MA_6375921g0010	-	6.44	12.41	165.8	-	-	-	-	-
MA_930826g0010	-	0	0	0.03	-	-	-	-	-
Gb_11546	15.64	35.42	17.72	25.05	31.42	15.21	-	-	-
Gb_25879	28.71	43.53	88.57	61.25	49.09	16.64	-	-	-
Smo111780	6.3	23.58	11.43	10.47	-	-	-	-	11.8
Smo97708	30.86	32.98	27.23	27.23	-	-	-	-	26.36
Pp3c13_15690V3.1	-	-	10.57	-	-	-	7.88	-	-
Pp3c15_5590V3.1	-	-	46.84	-	-	-	26.72	-	-
Pp3c3_21240V3.1	-	-	5.95	-	-	-	4.07	-	-
Mp7g09460.1	-	-	31.61	-	-	-	1.4	-	-
Mp8g17290.1	-	-	56.09	-	-	-	2.33	-	-

Expression patterns of plant *SAL* genes in root, flower, leaf, stem, female (ovaries, pistrils), seeds, male (pollen, anthers), apical meristem and root meristem. Expression of *SAL* genes of evolutionarily important lineages in eudicots (*Arabidopsis thaliana*, *Solanum lycopersicum*), monocots (*Oryza sativa*, *Zea mays*), Basal Angiosperm (*Amborella trichopoda*), Gymnosperms

(*Picea abies*, *Gingko biloba*), Lycophyte (*Selaginella moellendorffii*), Moss (*Physcomitrella patens*), and Liverwort (*Marchantia polymorpha*). Red indicating high expression, and blue indicating low expression, and missing data set as "-".

Supplementary Materials and Methods

Agrobacterium tumefaciens-mediated transformation of gametophytes

Stable genetic transformation of *C. richardii* plants was performed as described previously with modification (Bui et al., 2015; Bui et al., 2017; Withers et al., 2023). Briefly, a single colony of *Agrobacterium* carrying the desired vector was inoculated into 6 mL of LB with appropriate antibiotics (25 mg/L of Rifampin and 50 mg/L of Kanamycin) through 15 mL tube at 28°C (Jiang et al., 2022). The culture was allowed to grow for approximately 16 h. The cells were then collected by centrifugation of 6 mL of the culture at 5,000 rpm for 2 min. After that, *A. tumefaciens* was resuspended through the induction medium (IM) buffer with 200 μM acetosyringone (AS) (Michielse et al., 2008) to achieve OD600 = 0.8. Enzyme-treated gametophytes were incubated for 2 h at room temperature in petri dish with 5 mL enzyme treatment buffer, which was subsequently removed by three sequential rinses with sterile distilled water. Then, gametophytes were co-incubated with 3 mL of *Agrobacterium* culture for 1 h at room temperature in Petri dish with 3 mL enzyme treatment buffer. Ultimately, the gametophytes were plated on MS medium with IM buffer containing 200 μM AS for 72 h in the dark in the same chamber used to grow the gametophytes.

After IM incubated, gametophytes were washed three times through sterile distilled water containing 100 mg/L cefuroxime. Thereafter, gametophytes were grown at MS media with 5 mg/L of hygromycin and 100 mg/L of cefuroxime for 30 days to reach sexual maturity and form sporophytes. Every Petri dish (9 cm × 1.5 cm) includes 30–50 gametophytes. After that, the sporophytes were transferred to new MS media containing 20 mg/L of hygromycin and 100 mg/L of cefuroxime about 30 days for selecting positive plants. Sporophytes were then transplanted to pots containing a premium potting mix (Scotts Osmocote, Bella Vista, NSW Australia) with the cover to keep high humidity. Transgenic plants were later used for positive identification and gene expression when they had the third expanding frond. The CTAB protocol (Frangedakis et al., 2021) was used for extraction of *C. richardii* genomic DNA. Identification of transgenic plants was performed by hygromycin primers.

Medium

All medium were autoclaved at 121°C for 15 min.

MS:

For 1 L of media add the following to 850 mL of deionised water: 4.16 g Murashige and Skoog (MS) medium (Sigma), 20 g sucrose, and 0.5 g MES free acid. Adjust the pH to 5.8-6.0 once dissolved using 1 M NaOH (Sodium Hydroxide). 15 g agar for solid medium.

LB:

Weigh out 10 g tryptone, 5 g yeast extract, and 10 g NaCl (Sodium Chloride), and add to a 1 L Shott bottle with 1 L distilled water (1.5% agar for solid media). Once the reagents have fully dissolved, adjust the pH to 7.0-7.4 through 1 M NaOH solution.

Enzyme treatment buffer:

1.5% cellulase

0.5 M Mannitol

For 50 mL buffer: 4.555g Mannitol and 0.75g cellulase.

Induction Medium (IM buffer, liquid medium):

Mix 800 μL of 1.25 M **Potassium Phosphate Buffer**, 20 mL **MN Buffer**, 1 mL of 10 mg mL^{-1} **$CaCl_2$**, 1 mL of 1 mg mL^{-1} **$FeSO_4$**, 5 mL **IM-Salts**, 2 mL of 200 mg ml^{-1} **NH_4NO_3**, 10 mL of 50% **Glycerol**, 40 mL of 1 M **MES** and 1 mL of 200 mg/mL **Glucose** and make up the volume to 1000 mL. Autoclave at 121°C for 15 min. Cool to 55°C and add 1 mL of 200 mM acetosyringone. Mix vigorously.

1.25 M Potassium Phosphate Buffer: Dissolve 17 g KH_2PO_4 in 90 mL of distilled water and make up the volume to 100 mL. Dissolve 22 g K_2HPO_4 in 90 mL of distilled water and make up the volume to 100 mL. Add K_2HPO_4 solution dropwise to KH_2PO_4 solution until pH reaches the value of 4.8. Sterilize by autoclaving.

MN Buffer: Dissolve 30 g $MgSO_4{\cdot}7H_2O$ and 15 g NaCl in 900 mL of distilled water and make up the volume to 1000 mL. Filter-sterilize (0.2 μm) and store at room temperature.

10 mg/mL $CaCl_2$ $2H_2O$: Dissolve 1 g of $CaCl_2$ $2H_2O$ in 90 mL of distilled water and make up to 100 mL. Store at 4°C.

1 mg/mL $FeSO_4$ $7H_2O$: Dissolve 100 mg $FeSO_4 \cdot 7H_2O$ in 90 mL distilled water and make up to 100 mL. Store at 4°C.

IM-Salts: Dissolve 100 mg of each H_3BO_3, $ZnSO_4 \cdot 7H_2O$, $CuSO_4 \cdot 5H_2O$, $MnSO_4 \cdot H_2O$, and $Na_2MoO_4 \cdot 2H_2O$ in 900 mL of distilled water and make up the volume to 1000 mL. Filter-sterilize (0.2 µm) and store at 4°C.

200 mg/mL NH_4NO_3: Dissolve 20 g NH_4NO_3 in 70 mL of distilled water and makeup to 100 ml. Filter-sterilize (0.2 µm) and store at 4°C.

50% Glycerol: Add 50 mL glycerol to 50 mL of distilled water. Store at 4°C.

1 M MES: Dissolve 19.5 g MES in 90 mL of distilled water and stir at 50°C until MES is completely dissolved. Adjust pH to 5.5 with 5 M NaOH and make up the volume to 100 mL. Store at room temperature.

200 mg/mL Glucose: Dissolve 20 g glucose monohydrate in 90 mL of distilled water and stir at 50°C until glucose is completely dissolved. Make up to 100 mL and use immediately or store at 4°C.

References

Bui LT, Cordle AR, Irish EE, Cheng CL. Transient and stable transformation of *Ceratopteris richardii* gametophytes. BMC Res. Notes 2015: **8**: 214.

Bui LT, Pandzic D, Youngstrom CE, Wallace S, Irish EE, Szovenyi P, Cheng CL. A fern *AINTEGUMENTA* gene mirrors *BABY BOOM* in promoting apogamy in *Ceratopteris richardii*. Plant J. 2017: **90**: 122-132.

Jiang W, Tong T, Li W, Huang Z, Chen G, Zeng F, Riaz A, Amoanimaa-Dede H, Pan R, Zhang W, et al. Molecular evolution of plant 14-3-3 proteins and function of Hv14-3-3A in stomatal regulation and drought tolerance. Plant Cell Physiol. 2022: **63**: 1857-1872.

Marchant DB, Chen G, Cai S, Chen F, Schafran P, Jenkins J, Shu S, Plott C, Webber J, Lovell JT, et al. Dynamic genome evolution in a model fern. Nat. Plants 2022: **8**: 1038-1051.

Michielse CB, J Hooykaas PJ, J J van den Hondel CAM, J Ram AF. *Agrobacterium*-mediated transformation of the filamentous fungus *Aspergillus awamori*. Nat. Protoc. 2008: **3**: 1671-1678.

Withers KA, Falls K, Youngstrom CE, Nguyen T, DeWald A, Yarvis RM, Simons GP, Flanagan R, Bui LT, Irish EE, et al. A Ceratopteris EXCESS MICROSPOROCYTES1 suppresses reproductive transition in the fern vegetative leaves. Plant Sci. 2023: **335**: 111812.
